# Factors Associated With Psychological Outcomes Among Vaccinated and Unvaccinated Health Care Workers Against COVID-19 Infection in Bangladesh

**DOI:** 10.3389/fmed.2022.852922

**Published:** 2022-03-24

**Authors:** Md. Dhedharul Alam, Sujan Kumer Paul, Mahmuda Momi, Li Ni, Yi Xu

**Affiliations:** ^1^Department of Psychiatry, The First Affiliated Hospital, Zhejiang University School of Medicine, Hangzhou, China; ^2^The Key Laboratory of Mental Disorder Management in Zhejiang Province, Hangzhou, China; ^3^Department of Periodontology and Oral Pathology, Holy Family Red Crescent Medical College and Hospital, Dhaka, Bangladesh; ^4^Department of Restorative Dentistry, Faculty of Dentistry, University of Malaya, Kuala Lumpur, Malaysia; ^5^Department of Psychiatry, Fuyang Third Peoples Hospital, Hangzhou, China

**Keywords:** Bangladesh, COVID-19, health care workers, immunization, psychological outcomes, refusal, uptake

## Abstract

**Background:**

Vaccination of healthcare workers (HCWs) is recommended during the COVID-19 pandemic to reduce the risk of infection for themselves and their patients, as well as to encourage their patients to get immunized. The present study aimed to investigate the psychological outcomes and associated factors among vaccinated and unvaccinated HCWs against COVID-19 infection in Bangladesh.

**Methods:**

From March to August 2021, an online nationwide survey was conducted with a total of 2,038 Bangladeshi HCWs. The frequency of symptoms of general health problems, depression, anxiety, stress, post-traumatic stress disorder, insomnia, and loneliness was assessed using the Bangla versions of the GHQ-12, PHQ-2, GAD-2, PSS-4, PC-PTSD-5, ISI, and UCLA-LS scales, respectively.

**Results:**

Compared with unvaccinated HCWs (*n* = 1,058), vaccinated HCWs (*n* = 980) had a statistically significant lower prevalence of general health problems (16.7 vs. 59.1%), depression (15.6 vs. 31.9%), post-traumatic stress disorder (22.3 vs. 30.8%), insomnia (23.8 vs. 64.9%), and loneliness symptoms (13.9 vs. 21.8%). Among vaccinated HCWs, females were significantly associated with a higher risk of symptoms of general health problems (AOR, 2.71; 95% CI, 0.97–7.60), anxiety (AOR, 2.17; 95% CI, 1.14–4.13), and loneliness (AOR, 2.52; 95% CI, 1.11–5.73). Except for anxiety and post-traumatic stress disorder symptoms, participants living in urban areas had a significantly lower risk of all psychological outcomes (e.g., depression: AOR, 0.43; 95% CI, 0.27–0.67; stress: AOR, 0.64; 95% CI, 0.47–0.88). Respondents who were married were significantly less likely to experience symptoms of general health problems (AOR, 0.10; 95% CI, 0.02–0.39), depression (AOR, 0.31; 95% CI, 0.22–0.82), insomnia (AOR, 0.46; 95% CI, 0.20–1.03), and loneliness (AOR, 0.31; 95% CI, 0.10-0.92). Participants who worked as doctors were significantly less chance of experiencing symptoms of general health problems (AOR, 0.18; 95% CI, 0.08–0.37), depression (AOR, 0.51; 95% CI, 0.30–0.87), and anxiety (AOR, 0.54; 95% CI, 0.37–0.78). On the other hand, unvaccinated HCWs who were 18–29 years old and had <5 years of work experience were significantly associated with a higher risk of all psychological outcomes except anxiety and insomnia symptoms (e.g., depression among 18–29 years old: AOR, 1.83; 95% CI, 0.27–2.60; stress among those with <5 years of work experience: AOR, 2.37; 95% CI, 0.93–6.07). Participants who worked as nurses were significantly more likely to suffer from depression (AOR, 1.44; 95% CI, 0.84–2.46), anxiety (AOR, 1.42; 95% CI, 0.24–1.73), and stress (AOR, 1.55; 95% CI, 0.31–0.89) symptoms. Except for anxiety and stress symptoms, respondents who worked as frontline workers and provided direct care to infected patients were the significantly higher chance of experiencing all psychological outcomes (e.g., depression among who worked as frontline workers: AOR, 2.41; 95% CI, 0.23–3.73; insomnia among those who provide direct care to infected patients: AOR, 2.60; 95% CI, 0.34–3.06). Participants who were infected with COVID-19 had a significantly less chance of experiencing symptoms of general health problems (AOR, 0.89; 95% CI, 0.65–1.22), depression (AOR, 0.66; 95% CI, 0.48–0.92), and anxiety (AOR, 0.63; 95% CI, 0.46–0.87).

**Conclusions:**

To control the infection and improve psychological outcomes, this study suggests emphasizing the vaccinated to unvaccinated HCWs as soon as possible. They also required special attention, health-related education, and psychological support.

## Introduction

The Coronavirus Disease 2019 (COVID-19) has now spread throughout the world. Since the commencement of the COVID-19 pandemic in 2019, around 225 countries and 215.7 million people have been afflicted with the virus, which has killed about 4.4 million people (as of August 29, 2021) (1). This unprecedented global epidemic poses a severe challenge to local healthcare systems, with a growing number of daily cases and death counts related to COVID-19. Healthcare workers (HCWs) are more vulnerable to COVID-19 than the general population, particularly those exposed to suspected and confirmed cases, due to the high risk of infection, insufficient protection and disease management experience, heavy workload, substantial lifestyle adjustments, quarantine, and lower social support (2–4). These variables raise the risk of psychological issues among HCWs, including depression, anxiety, insomnia, fear, and suicide, all of which can have a severe impact on work productivity and long-term well-being (5–7).

However, Sanghera et al. (8) conducted a meta-analysis of 44 studies involving 69,499 HCWs, reporting high rates of indications of depression (13.5–44.7%), anxiety (12.3–35.6%), stress (5.2–32.9%), post-traumatic stress disorder (7.4–37.4%), insomnia (33.8–36.1%) and burnout (3.1–43.0%) among HCWs during the COVID-19 outbreak. Another meta-analysis of the effects of SARS, MARS, and COVID-19 on HCWs' physical and mental health found that general health concerns (62.5%), depression (26.3%), anxiety (29.0%), post-traumatic stress disorder (20.7%), insomnia (37.9%), psychological distress (37.8%), fear (43.7%), burnout (34.4%), somatization (16.1%), and stigmatization feelings (14.0%) (9). Bangladesh, where the current study was done, is a South Asian country where COVID-19 has significantly impacted its healthcare system (10). The first COVID-19 case was reported in Bangladesh on March 8, 2020 (11), and as of August 31, 2021, the country had 1.4 million verified COVID-19 cases and 26,195 deaths (12). Bangladesh reported the first death on April 15, 2020, and a nurse on May 30, 2020. Approximately 9,394 healthcare providers had been infected with the virus on August 29, 2021, with 186 of them dying (Supplementary Figures 1, 2) (13, 14). A study examining the impact of the COVID-19 pandemic on Bangladeshi HCWs found that the prevalence of depression, anxiety, insomnia, and loneliness among HCWs were 44, 78, 89, and 87%, respectively (15).

Vaccines are one of the most effective strategies for preventing COVID-19 infection, as well as its consequences and complications (16). Since the first COVID-19 vaccination human clinical trial began on March 3, 2020 (17), 33 vaccines had progressed to stage 3 clinical trials, with 22 vaccines approved in 192 countries by August 31, 2021 (18). More than 5 billion doses of the vaccine were already administered globally as of August 31, 2021 (1). On January 27, 2021, Bangladesh began providing COVID-19 vaccines, with bulk vaccination starting on February 7, 2021, and the second dosage starting on April 8, 2021 (19). As of August 31, 2021, the number of first doses administered in Bangladesh is 18,489,742, and the number of second doses administered is 8,045,469 (Supplementary Figure 3) (12). Ideally, a high enough percentage of the population will be immunized, safeguarding those who aren't, a process known as “herd immunity.” It has been estimated between 55 to 82% of populations would need to be vaccinated to reach herd immunity for COVID-19, depending on varying biological, environmental, socio-behavioral factors and infection rates within each country (20).

Given the significant increase in anxiety and depressive symptoms linked to the COVID-19 pandemic's stress (21), it is plausible to believe that vaccination could lead to reduced anxiety and depressive symptoms. However, it is not known whether the psychological status would be affected after COVID-19 vaccination. One study showed that COVID-19 vaccination could positively correlate with COVID-19-related anxiety and fears among 1,779 adults in Germany (22), while another study indicated that psychological stress levels after getting vaccinated significantly decreased among the public in China (23). In addition, a cross-sectional survey of 363 HCWs in Turkey indicated that COVID-19 vaccination was not linked to secondary traumatic stress, anxiety, and depression symptoms among HCWs (24). As a result, it's critical to look into how this COVID-19 immunization affects mental health, particularly among HCWs. However, there have been no studies on the psychological outcomes of COVID-19 vaccination on both vaccinated and unvaccinated HCWs in Bangladesh yet. Therefore, we conducted a cross-sectional survey to assess the factors associated with psychological outcomes among vaccinated and unvaccinated HCWs against SARS-CoV-2 infection in Bangladesh. This study looked into the prevalence of general health problems, depression, anxiety, stress, post-traumatic stress disorder, insomnia, and loneliness among vaccinated and unvaccinated HCWs against SARS-CoV-2 infection in Bangladesh and explored its contributing factors.

Based on these considerations, this study had three objectives. First, we sought to determine the prevalence of general health problems, depression, anxiety, stress, post-traumatic stress disorder, insomnia, and loneliness among vaccinated and unvaccinated HCWs against SARS-CoV-2 infection in Bangladesh. Second, we sought to identify a difference in the prevalence of general health problems, depression, anxiety, stress, post-traumatic stress disorder, insomnia, and loneliness symptoms among vaccinated and unvaccinated HCWs in Bangladesh. Third, we sought to explore which socio-demographic and clinical factors could significantly predict psychological outcomes in the group of vaccinated and unvaccinated HCWs against SARS-CoV-2 infection in Bangladesh. Based on these objectives, we hypothesized that vaccinated HCWs had a lower prevalence of psychological outcomes against SARS-CoV-2 infection in Bangladesh than unvaccinated HCWs. This research will add to our understanding of SARS-CoV-2 vaccination and mental health and assist governments and policymakers in developing an effective vaccine campaign to achieve vaccination coverage and herd immunity among HCWs and the public during the SARS-CoV-2 outbreak.

## Materials and Methods

### Study Design

The study was approved by the Institutional Ethical Review Board (IERB) of the Holy Family Red Crescent Medical College and Hospital, Dhaka, Bangladesh (Approval No: IERB/36) and the Ethics Committee of the First Affiliated Hospital, Zhejiang University School of Medicine before it began. Before the participants started the questionnaire, they had to give their informed consent online. Between March and August of 2021, a cross-sectional online study was administered. The data was obtained online using Google Forms and the Bangla language. The two research assistants sent the survey link by e-mail, Facebook, Viber, WhatsApp, Imo, and other social media platforms. They were invited to fill out the form and share the link with their networks to reach more people. They used the convenient and snowball method to circulate the survey link throughout their professional and social networks. Participants were told that taking part in the study was completely voluntary, and they were urged to share the survey link with their friends or acquaintances once it was completed. All participants were assured of their data's privacy and confidentiality, as well as information on the study's goal, protocol, and their right to have their data removed at any time. The current study received a total of 2,067 responses at the onset. After screening, 29 responses were eliminated due to missing information, not being fully vaccinated, and being outside of Bangladesh. Finally, responses from 2,038 HCWs were included in this study. Nine hundred and eighty HCWs had been vaccinated, and 1,058 had not. Vaccinated means they had fully dose vaccinated. The following were the criteria for inclusion: (1) be at least 18 years old, (2) living in Bangladesh at the time of the COVID-19, (3) willingness to engage in this study via online informed consent, (4) completion of the whole questionnaire, and (5) no history of mental health problems.

### Participants

The sample size was calculated using OpenEpi software. A previous study on the SARS-CoV-2 outbreak in Bangladesh found that 50% of HCWs had psychological problems (25). This 50% proportion would provide maximum variance and sample size. At 95% confidence level, 80% power, and 1.5 design effect, we arrived at the sample size of 576. The current study inflated our sample by 10% to account for non-response data, so the final sample size required was 634 participants for each group.

### Measurements

#### Demographic Information

The participant's sex (male, female, or not interested), age (18–29, 30–39, 40–49, or 50 years), residence (urban and rural), the status of marriage, whether or not they had children, and educational level were self-reported demographic information. Participants were also asked working position (doctor, nurse, medical technician, hospital workers or other), work types (frontline or second-line), employment titles (senior, intermediate, junior, new or other), work experiences (≤5, 6-10, 11–19, or ≥20 years), socioeconomic status (lower, middle or upper class), living with family, and smoking habit. In addition, this study also investigated whether participants had provided direct care to infected patients, whether they had been infected with COVID-19, whether anyone in their family, friends, or colleagues had been infected with COVID-19, and whether anyone in their family, friends, or colleagues had died from COVID-19.

#### General Health Questionnaire

The 12-item validated Bangla version of the General Health Questionnaire (GHQ-12) (26, 27) evaluates mental health status on a four-point Likert scale, with “1” defining never and “4” defining frequently. For a full score of 0–12, each item can be assigned a value of 0 (if option 1 or 2) or 1 (if options 3 and 4). The overall score of ≥3 indicated that the person's mental health status was terrible. In this study, the internal consistency was α = 0.81.

#### Patient Health Questionnaire

The two-item validated Bangla version of the Patient Health Questionnaire (PHQ-2) (28–30) evaluates depression symptoms rated on a four-point Likert scale, with “1” defining never and '4' defining almost every day. The overall value of ≥3 is suggested to indicate a likely diagnosis of significant depression. In this study, the internal consistency was α = 0.76.

#### Generalized Anxiety Disorder Scale

The two-item validated Bangla version of the Generalized Anxiety Disorder scale (GAD-2) (31, 32) evaluates anxiety symptoms on a four-point Likert scale, with “1” defining never and “4” defining almost every day. The overall score of ≥3 is proposed as revealing a probable anxiety disorder diagnosis. The internal consistency was α = 0.77.

#### Perceived Stress Scale

The four-item validated Bangla version of the Perceived Stress Scale (PSS-4) (33–35) evaluates stress symptoms on a four-point Likert scale, with “1” defining never and “4” defining always. A quartile split was used because no official cut-off for the PSS-4 scale was available. In this study, the internal consistency was α = 0.72.

#### Primary Care PTSD Screen for DSM-5

The Bangla version of the Primary Care PTSD Screen for DSM-5 (PC-PTSD-5) (36) evaluates post-traumatic stress disorder symptoms over the past month by asking five binary questions about re-experiencing, avoidance, physiological reactions, emotional numbness, and trauma-distorted guilt and blame thoughts. This scale was previously used in a Bangladeshi study (37). The total score ranges from 1 to 5, with a 3 as the cut-off value. In this study, the internal consistency was α = 0.71.

#### Insomnia Severity Index

The seven-item validated Bangla version of the Insomnia Severity Index (ISI) (38, 39) evaluates the severity of insomnia on a five-point Likert scale, with “0” defining no problem and “4” defining a major problem. An overall score of ≥8 indicates possible insomnia symptoms in this investigation. The internal consistency was α = 0.72.

#### University of California, Los Angeles, Loneliness Scale

The three-item validated Bangla version of the University of California, Los Angeles, Loneliness Scale (UCLA-LS) (40, 41) evaluates loneliness symptoms on a three-point Likert scale, with “1” defining rarely and “3” defining frequently. Participants who received a score of ≥6 were considered to be lonely to a high degree. In this study, the internal consistency was α = 0.75.

#### Oslo Social Support Scale

The Bangla version of the three-item Oslo Social Support Scale (OSSS-3) (42) was also used to evaluate respondents' social support. The raw scores were added together to create a sum index, ranging from 3 to 14. Social support was labeled as poor, moderate, or strong based on a score of 3-8, 9-11, or 12-14. In this study, the internal consistency was α = 0.75.

The PC-PTSD-5 and OSSS-3 scales were first sent to three experts in medicine, public health, and epidemiology, who translated the English version into Bangla and then back into English to ensure internal consistency, validity, and acceptable reliability (43). The scales were then piloted with 30 potential respondents from various categories to ensure that the language in the final version was perfect. The tools used in the pilot study received no corrections or suggestions from respondents regarding the contents developed in Bangla.

### Statistical Analysis

The statistical analyses were run by SPSS version 20.0, and figures were prepared in GraphPad Prism version 9. Categorical data was represented using numbers and percentages. To compare categorical variable variations between groups, Chi-square tests were used. The Kolmogorov–Smirnov test, the Shapiro–Wilk test, and normal Q-Q plots were used to determine the data's normality. The median of the interquartile range (IQR) of data from non-normal distributions was shown. When comparing non-normally distributed data between two groups, the Mann–Whitney *U*-test was used, and when comparing data between more than two groups, the Kruskal–Wallis-test was used. Spearman correlations were used to compare the psychological outcomes of vaccinated and unvaccinated HCWs. In addition, binary logistic regression analysis was used to look into potential predictors of psychological outcomes in both groups. The model fitness test was checked using the Hosmer and Lemeshow goodness of fit test. All of the variables were added in the univariate analysis and then the multivariate analysis only included the significant variables in the univariate analysis. For a single predictor, univariate analysis expressed as crude odds ratio (COR) was used, while multivariate analysis expressed as adjusted odds ratio (AOR) was used for multiple predictors, and all psychological outcomes were considered dependent variables. All analyses were conducted at a 95% confidence level, with *p*-values <0.05 considered significant.

## Results

### Sample Characteristics

Finally, 2,038 HCWs were enrolled in our study, with 980 (48.1%) being vaccinated and 1,058 (51.9%) being unvaccinated. The characteristics of the study respondents are shown in Table 1. Vaccinated HCWs were significantly more likely to be younger (41.8 vs. 39.3%, *p* < 0.01), doctors (42.9 vs. 22.3%, *p* < 0.01), frontline workers (62.6 vs. 47.1%, *p* < 0.01), junior HCWs (48.4 vs. 38.1%, *p* < 0.01), with <5 years of work experience (52.8 vs. 45.8%, *p* < 0.01), from a middle-class socioeconomic status (59.6 vs. 53.8%, *p* < 0.01), providing direct service to infected patients (68.6 vs. 44.0%, *p* < 0.01), infected with COVID-19 (45.1 vs. 23.8%, *p* < 0.01), and with moderate social support (57.3 vs. 34.8%, *p* < 0.01) than unvaccinated HCWs. On the other hand, unvaccinated HCWs were significantly more male (52.7 vs. 47.2%, *p* < 0.01), married (62.1 vs. 58.2%, *p* < 0.01), had a post-graduate degree (54.9 vs. 45.0%, *p* < 0.01), lived with family (70.0 vs. 51.9%, *p* < 0.01), had family members, friends, or colleagues infected with COVID-19 (57.8 vs. 30.6%, *p* < 0.01) and died from it (33.3 vs. 23.6%, *p* < 0.01) than vaccinated HCWs. Moreover, there were no significant differences between the vaccinated and unvaccinated HCWs in terms of residence (*p* = 0.41), having children (*p* = 0.63), and smoking habits (*p* = 0.25).

**Table 1:** Sociodemographic characteristics in vaccinated and unvaccinated health care workers against COVID-19 infection.

**Characteristics**	**Total** **(n = 2038)**	**Vaccinated health care workers** **(n = 980)**	**Unvaccinated health care workers (n = 1058)**	**p value**
	**No. (%)**	**No. (%)**	**No. (%)**	
**Sex**				
Male	1021 (50.1)	463 (47.2)	558 (52.7)	<0.01
Female	953 (46.8)	461 (47.0)	492 (46.5)	
Not interested	64 (3.1)	56 (5.7)	8 (0.8)	
**Age, Y**				
18–29	826 (40.5)	410 (41.8)	416 (39.3)	<0.01
30–39	612 (30.0)	257 (26.2)	355 (33.6)	
40–49	407 (20.0)	205 (20.9)	202 (18.1)	
≥50	193 (9.5)	108 (11.0)	85 (8.0)	
**Residence**				
Urban	1483 (72.8)	705 (71.9)	778 (73.5)	0.41
Rural	555 (27.2)	275 (28.1)	280 (26.5)	
**Marital status**				
Single	608 (29.8)	342 (34.9)	266 (25.1)	<0.01
Married	1227 (60.2)	570 (58.2)	657 (62.1)	
Divorced/separated/widowed	203 (10.0)	68 (6.9)	135 (12.8)	
**Having children**				
Yes	1049 (51.5)	499 (50.9)	550 (52.0)	0.63
No	989 (48.5)	481 (49.1)	508 (48.0)	
**Education level**				
Bachelor (MBBS) or lower degree	625 (30.7)	338 (34.5)	287 (27.1)	
Post-graduate degree	1022 (50.1)	441 (45.0)	581 (54.9)	<0.01
Doctoral degree	383 (18.8)	195 (19.9)	188 (17.8)	
Other	8 (0.4)	6 (0.6)	2 (0.2)	
**Working position**				
Doctor	656 (32.2)	420 (42.9)	236 (22.3)	<0.01
Nurse	159 (7.8)	69 (7.0)	90 (8.5)	
Medical technician	249 (12.2)	79 (8.1)	170 (16.1)	
Hospital workers	303 (14.9)	99 (10.1)	204 (19.3)	
Other	671 (32.9)	313 (31.9)	358 (33.8)	
**Work types**				
Frontline	1111 (54.5)	613 (62.6)	498 (47.1)	<0.01
Second-line	927 (45.5)	367 (37.4)	560 (52.9)	
**Employment titles**				
Senior	311 (15.3)	154 (15.7)	157 (14.8)	<0.01
Intermediate	473 (23.2)	200 (20.4)	273 (25.8)	
Junior	877 (43.0)	474 (48.4)	403 (38.1)	
New	366 (18.0)	143 (14.6)	223 (21.1)	
Other	11 (0.5)	9 (0.9)	2 (0.2)	
**Work experiences, Y**				
≤5	1002 (49.2)	517 (52.8)	485 (45.8)	<0.01
6–10	387 (19.0)	142 (14.5)	245 (23.2)	
11–19	422 (20.7)	187 (19.1)	235 (22.2)	
≥20	227 (11.1)	134 (13.7)	93 (8.8)	
**Socio economic status**				
Lower class	591 (29.0)	286 (29.2)	305 (28.8)	<0.01
Middle class	1153 (56.6)	584 (59.6)	569 (53.8)	
Upper class	294 (14.4)	110 (11.2)	184 (17.4)	
**Living with family**				
Yes	1250 (61.3)	509 (51.9)	741 (70.0)	<0.01
No	788 (38.7)	471 (48.1)	317 (30.0)	
**Smoking habit**				
Yes	613 (30.1)	283 (28.9)	330 (31.2)	0.25
No	1425 (69.9)	697 (71.1)	728 (68.8)	
**Providing direct service to infected patients**				
Yes	1138 (55.8)	672 (68.6)	466 (44.0)	<0.01
No	900 (44.2)	308 (31.4)	592 (56.0)	
**Have you been infected with COVID-19?**				
Yes	694 (34.1)	442 (45.1)	252 (23.8)	<0.01
No	1344 (65.9)	538 (54.9)	806 (76.2)	
**Have any of your family members, friends, or colleagues been infected with the COVID-19?**				
Yes	912 (44.7)	300 (30.6)	612 (57.8)	<0.01
No	1,126 (55.3)	680 (69.4)	446 (42.2)	
**Have any of your family members, friends, or colleagues died of the COVID-19?**				
Yes	583 (28.6)	231 (23.6)	352 (33.3)	<0.01
No	1,455 (71.4)	749 (76.4)	706 (66.7)	
**Social support**				
Poor	807 (39.6)	219 (22.3)	588 (55.6)	<0.01
Moderate	930 (45.6)	562 (57.3)	368 (34.8)	
Strong	301 (14.8)	199 (20.3)	102 (9.6)	

### Scores of Psychological Outcomes

When compared to unvaccinated HCWs, vaccinated HCWs had significantly lower median of the interquartile range (IQR) of scores for general health problems (2.0 [0–2.0] vs. 4.0 [2.0–6.0]; *p* < 0.01), depression (1.0 [1.0–2.0] vs. 3.0 [2.0-5.0]; *p* < 0.01), post-traumatic stress disorder (1.0 [1.0–2.0] vs. 3.0 [1.0-4.0]; *p* < 0.01), insomnia (3.0 [4.0-7.0] vs. 6.0 [5.0–11.0]; *p* < 0.01), and loneliness (1.0 [3.0–4.0] vs. 2.0 [3.0–5.0]; *p* < 0.01) symptoms, but significantly higher median of the interquartile range (IQR) of scores for anxiety (2.0 [1.0–3.0] vs. 1.0 [2.0–3.0]; *p* < 0.01), and stress (7.0 [8.0–15.0] vs. 5.0 [8.0–13.2]; *p* < 0.01) symptoms (Table 2).

**Table 2:** The median of the interquartile range (IQR) of psychological outcome scores in vaccinated and unvaccinated health care workers against COVID-19 infection.

**Psychological** **outcomes**	**Total** **score**	**Vaccinated health care workers**	**Unvaccinated health care workers**	***P*-** **value**
	**Median (IQR)**	**Median (IQR)**	**Median** **(IQR)**	
General health problems	4.0 (1.0–5.0)	2.0 (0–2.0)	4.0 (2.0–6.0)	<0.01
Depression symptoms	1.0 (1.0–2.0)	1.0 (1.0–2.0)	3.0 (2.0–5.0)	<0.01
Anxiety symptoms	1.0 (2.0–3.0)	2.0 (1.0–3.0)	1.0 (2.0–3.0)	<0.01
Stress symptoms	6.0 (8.0–14.0)	7.0 (8.0–15.0)	5.0 (8.0–13.2)	<0.01
Post-traumatic stress disorder symptoms	2.0 (1.0–3.2)	1.0 (1.0–2.0)	3.0 (1.0–4.0)	<0.01
Insomnia symptoms	6.0 (5.0–11.0)	3.0 (4.0–7.0)	6.0 (5.0–11.0)	<0.01
Loneliness symptoms	2.0 (3.0–5.0)	1.0 (3.0–4.0)	2.0 (3.0–4.0)	<0.01

*IQR, Interquartile range*.

### Prevalence of Psychological Outcomes

The prevalence of psychological outcomes among vaccinated and unvaccinated health care workers against COVID-19 infection are shown in Table 3. The prevalence rates of symptoms of general health problems, depression, anxiety, stress, post-traumatic stress disorder, insomnia, and loneliness symptoms among vaccinated HCWs were 16.7, 15.6, 24.8, 34.7, 22.3, 23.8, and 13.9%, respectively. On the other hand, the prevalence rates of symptoms of general health problems, depression, anxiety, stress, post-traumatic stress disorder, insomnia, and loneliness symptoms among unvaccinated HCWs were 59.1, 31.9, 26.1, 35.0, 30.8, 64.9, and 21.8%, respectively. However, vaccinated HCWs had a significantly lower prevalence rates of general health problems (16.7 vs. 59.1%, *p* < 0.01), depression (15.6 vs. 31.9%, *p* < 0.01), post-traumatic stress disorder (22.3 vs. 30.8%, *p* < 0.01), insomnia (23.8 vs. 64.9%, *p* < 0.01), and loneliness symptoms (13.9 vs. 21.8%, *p* < 0.01) compared to unvaccinated HCWs. Moreover, the vaccinated and unvaccinated HCWs did not differ significantly on anxiety (24.8 vs. 26.1%, *p* = 0.50) and stress (34.7 vs. 35.0%, *p* = 0.89) symptoms.

**Table 3:** The prevalence of psychological outcomes among vaccinated and unvaccinated health care workers against COVID-19 infection.

**Measure**	**Total** **(*n* = 2,038)**	**Vaccinated health care workers** **(*n* = 980)**	**Unvaccinated health care workers** **(*n* = 1,058)**	***P*-** **value**
	**No. (%)**	**No. (%)**	**No. (%)**	
**General health problems**				
Yes	789 (38.7)	164 (16.7)	625 (59.1)	<0.01
No	1,249 (61.3)	816 (83.3)	433 (40.9)	
**Depression symptoms**				
Yes	491 (24.1)	153 (15.6)	338 (31.9)	<0.01
No	1,547 (75.9)	827 (84.4)	720 (68.1)	
**Anxiety symptoms**				
Yes	594 (29.1)	243 (24.8)	276 (26.1)	0.50
No	1,444 (70.9)	737 (75.2)	782 (73.9)	
**Stress symptoms**				
Yes	710 (34.8)	340 (34.7)	370 (35.0)	0.89
No	1,328 (65.2)	640 (65.3)	688 (65.0)	
**Post-traumatic stress disorder symptoms**				
Yes	545 (26.7)	219 (22.3)	326 (30.8)	<0.01
No	1,493 (73.3)	761 (77.7)	732 (69.2)	
**Insomnia symptoms**				
Yes	920 (45.1)	233 (23.8)	687 (64.9)	<0.01
No	1,118 (54.9)	747 (76.2)	371 (35.1)	
**Loneliness symptoms**				
Yes	367 (18.0)	136 (13.9)	231 (21.8)	<0.01
No	1,671 (82.0)	844 (86.1)	827 (78.2)	

### Correlations of Psychological Outcomes

Spearman's correlations of psychological outcomes among vaccinated and unvaccinated HCWs are shown in Table 4. In the vaccinated HCWs, there was a positive correlation between general health problems scores and depression (*r*_*s*_ = 0.208, *p* < 0.01), insomnia (*r*_*s*_ = 0.285, *p* < 0.01), and loneliness (*r*_*s*_ = 0.138, *p* < 0.01) scores, but a negative correlation with post-traumatic stress disorder (*r*_*s*_ = 0.135, *p* < 0.01) scores. Moreover, depression scores were positively linked to insomnia (*r*_*s*_ = 0.153, *p* < 0.01) and loneliness (*r*_*s*_ = 0.139, *p* < 0.01) scores, but negatively related to post-traumatic stress disorder (r_s_ = 0.071, *p* < 0.05) scores. Furthermore, there was a negative relationship between anxiety and post-traumatic stress disorder (r_s_ = 0.168, *p* < 0.01) scores, as well as anxiety and insomnia (r_s_ = 0.073, *p* < 0.05) scores. In addition, we found a positive link between insomnia and loneliness scores (r_s_ = 0.147, *p* < 0.01).

**Table 4:** Spearman's correlations of psychological outcomes among vaccinated and unvaccinated health care workers against COVID-19 infection.

**Health care workers**	**Psychological outcomes**	**1**	**2**	**3**	**4**	**5**	**6**	**7**
Vaccinated health care workers	1	1.00						
	2	0.208^**^	1.00					
	3	0.055	0.000	1.00				
	4	0.052	0.031	0.000	1.00			
	5	−0.135^**^	−0.071^*^	−0.168^**^	0.032	1.00		
	6	0.285^**^	0.153^**^	−0.073^*^	0.043	0.037	1.00	
	7	0.138^**^	0.139^**^	0.054	0.062	0.000	0.147^**^	1.00
Unvaccinated health care workers	1	1.00						
	2	0.127^**^	1.00					
	3	0.024	0.063^*^	1.00				
	4	−0.037	0.019	0.039	1.00			
	5	0.147^**^	0.047	0.023	−0.051	1.00		
	6	0.349^**^	0.147^**^	0.090^**^	−0.009	0.190^**^	1.00	
	7	0.079^*^	−0.013	−0.002	0.023	0.236^**^	0.078^*^	1.00

* *p < 0.05*,

** *p < 0.01. 1 General health problems, 2 Depression, 3 Anxiety, 4 Stress, 5 Post-traumatic stress disorder, 6 Insomnia, and 7 Loneliness*.

In the unvaccinated HCWs, general health problems scores were positively linked to depression (*r*_*s*_ = 0.127, *p* < 0.01), post-traumatic stress disorder (*r*_*s*_ = 0.147, *p* < 0.01), insomnia (*r*_*s*_ = 0.349, *p* < 0.01), and loneliness (*r*_*s*_ = 0.079, *p* < 0.05) scores. Moreover, there was a significant positive correlation between depression and anxiety (*r*_*s*_ = 0.063, *p* < 0.05), along with depression and insomnia (*r*_*s*_ = 0.147, *p* < 0.01) scores. Only a significant positive relationship existed between anxiety and insomnia (*r*_*s*_ = 0.090, *p* < 0.01) scores. Furthermore, there was a positive relationship between post-traumatic stress disorder and insomnia (r_s_ = 0.190, *p* < 0.01), as well as post-traumatic stress disorder and loneliness (r_s_ = 0.236, *p* < 0.01) scores. In addition, the study discovered a positive link between insomnia and loneliness (r_s_ = 0.078, *p* < 0.05) scores.

### Risk Factors of Psychological Outcomes

The results of the univariate logistic regression analysis of factors associated with psychological outcomes among vaccinated and unvaccinated health care workers against COVID-19 infection are presented in Supplementary Table 1. The variables found to be significant in the univariate logistic regression analysis were included in the multivariate analysis. The multivariate logistic regression analysis (Supplementary Table 2) showed that among vaccinated HCWs, females were significantly associated with a higher risk of symptoms of general health problems (AOR, 2.71; 95% CI, 0.97–7.60), anxiety (AOR, 2.17; 95% CI, 1.14–4.13), and loneliness (AOR, 2.52; 95% CI, 1.11–5.73) compared to males. Except for anxiety and post-traumatic stress disorder symptoms, participants living in urban areas had a significantly lower risk of all psychological symptoms than those living in rural areas (general health: AOR, 0.15; 95% CI, 0.09–0.25; depression: AOR, 0.43; 95% CI, 0.27–0.67; stress: AOR, 0.64; 95% CI, 0.47–0.88; insomnia: AOR, 0.41; 95% CI, 0.29-0.59; and loneliness: AOR, 0.29; 95% CI, 0.19-0.44). Respondents who were married were significantly less likely to experience symptoms of general health problems (AOR, 0.10; 95% CI, 0.02-0.39), depression (AOR, 0.31; 95% CI, 0.22–0.82), insomnia (AOR, 0.46; 95% CI, 0.20-1.03), and loneliness (AOR, 0.31; 95% CI, 0.10–0.92) than divorced, separated, or widowed respondents. Participants who worked as doctors were significantly less likely to experience symptoms of general health problems (AOR, 0.18; 95% CI, 0.08–0.37), depression (AOR, 0.51; 95% CI, 0.30–0.87), and anxiety (AOR, 0.54; 95% CI, 0.37–0.78) compared to other working positions.

On the other hand, unvaccinated HCWs who were 18–29 years old and had <5 years of work experience were significantly associated with a higher risk of all psychological outcomes except anxiety and insomnia symptoms (e.g., depression among 18–29 years old: AOR, 1.83; 95% CI, 0.27–2.60; stress among those with <5 years of work experience: AOR, 2.37; 95% CI, 0.93–6.07). Participants who worked as nurses were significantly more likely to suffer from depression (AOR, 1.44; 95% CI, 0.84–2.46), anxiety (AOR, 1.42; 95% CI, 0.24–1.73), and stress (AOR, 1.55; 95% CI, 0.31–0.89) than those who worked in other positions. Except for anxiety and stress symptoms, respondents who worked as frontline workers and provided direct care to infected patients were the significantly higher chance of experiencing all psychological outcomes (e.g., depression among who worked as frontline workers: AOR, 2.41; 95% CI, 0.23–3.73; insomnia among those who provide direct care to infected patients: AOR, 2.60; 95% CI, 0.34–3.06). Respondents who were infected with COVID-19 had a significantly less chance of experiencing symptoms of general health problems (AOR, 0.89; 95% CI, 0.65–1.22), depression (AOR, 0.66; 95% CI, 0.48–0.92), and anxiety (AOR, 0.63; 95% CI, 0.46–0.87) when compared to those who were not.

## Discussion

This is the first nationwide study in Bangladesh that has evaluated the factors associated with psychological outcomes among vaccinated and unvaccinated HCWs against COVID-19 infection. A total of 2,038 HCWs were enrolled in this study (980 being vaccinated and 1,058 being unvaccinated). Our study found that the prevalence rates of general health problems, depression, anxiety, stress, post-traumatic stress disorder, insomnia, and loneliness symptoms among vaccinated HCWs were 16.7, 15.6, 24.8, 34.7, 22.3, 23.8, and 13.9%, respectively. On the other hand, the prevalence rates of general health problems, depression, anxiety, stress, post-traumatic stress disorder, insomnia, and loneliness symptoms among unvaccinated HCWs were 59.1, 31.9, 26.1, 35.0, 30.8, 64.9, and 21.8%, respectively. However, our study revealed that vaccinated HCWs showed statistically significant differences in lower prevalence rates of general health problems, depression, post-traumatic stress disorder, insomnia, and loneliness symptoms than unvaccinated HCWs. Moreover, no statistically significant differences in anxiety and stress symptoms between both groups were found. Similarly, as compared to unvaccinated HCWs, vaccinated HCWs had considerably lower median (IQR) scores on general health problems, depression, post-traumatic stress disorder, insomnia, and loneliness symptoms. According to Spearman's correlations, among vaccinated HCWs, there was a positive correlation between general health problems scores and depression, insomnia, and loneliness scores, but a negative correlation with post-traumatic stress disorder scores. In the unvaccinated HCWs, general health problems scores were positively linked to depression, post-traumatic stress disorder, insomnia, and loneliness scores.

This research indicated that vaccinated HCWs had a lower prevalence of psychological outcomes than unvaccinated HCWs against the COVID-19 outbreak in Bangladesh. These findings paralleled a study conducted in the United States among 300 HCWs, which revealed that vaccination against COVID-19 improved HCWs' physical and mental health (44). Another study conducted in China reported that the COVID-19 vaccine could improve the mental health status of vaccinated individuals (45). Moreover, Chen et al. (46) study were done between January 6-June 7, 2021, reported that being vaccinated for SARS-CoV-2 was associated with lower odds of depressive symptoms than those not vaccinated. Furthermore, our findings were also consistent with another study, which showed that human papillomavirus (HPV) vaccination might relieve the depression of vaccinated individuals (47). Based on the information presented above, our hypotheses were partially confirmed. The current study discovered many factors linked to both vaccinated and unvaccinated HCWs.

Our findings showed that females vaccinated HCWs were significantly associated with a higher risk of symptoms of general health problems, anxiety, and loneliness compared to males. This finding was consistent with previous research, which found that female HCWs were poorer psychological outcomes than males before the vaccination program (10, 48–50) and that females were more accepting of COVID-19 vaccination than males (51, 52). This study revealed that except for anxiety and post-traumatic stress disorder symptoms, participants living in urban areas had a significantly lower risk of all psychological symptoms among vaccinated HCWs. These findings were in line with prior Bangladeshi studies (15, 53, 54), which claimed that HCWs working in urban areas had a higher rate of psychological outcomes. In a cross-sectional survey of 3,646 adults in Bangladesh, Avedin et al. (55) discovered that 81% of urban participants wanted to be vaccinated. Similar studies also found that participants who lived in a city were similarly more likely to pay for and take the COVID-19 vaccine (56, 57). In Bangladesh, urban areas may have higher rates of infection and mortality among HCWs and the general population than rural areas (53). Most doctors are located in Dhaka and major cities (58). HCWs working in COVID-19 and non-COVID settings face a high workload, constant exposure, infection risk, ethical decisions about rationing resources among patients, and safety concerns for family members (49, 53). As a result, the concerned authority should pay particular attention and care to vaccinated HCWs from urban areas during this or future pandemics.

Our findings revealed that being married was a common risk factor for general health problems, depression, insomnia, and loneliness symptoms among vaccinated HCWs, which contradicts a recent national cross-sectional study involving 453,167 participants in the United States, which found that widowed, divorced, or separated people have a stronger association between SARS-CoV-2 vaccination and reduced depression and anxiety symptoms (46). However, our findings are in line with previous research, which found that being married is a common risk factor for adverse psychological outcomes (53, 59). However, in a recently published study of HCWs affected by the COVID-19 pandemic, married HCWs reported higher scores in vicarious traumatization symptoms than unmarried HCWs (60). It could be the reason for married HCWs having more occupational exhaustion and family responsibilities than unmarried HCWs.

The current study discovered that being a doctor is an independent risk factor for general health problems, depression, and anxiety symptoms among vaccinated HCWs, which was in agreement with prior studies that found doctors to be more vulnerable to COVID-19 (37, 61, 62). Similarly, in a study of 450 HCWs in Ethiopia, Angelo et al. (63) discovered that physicians were nearly fifteen times more likely than other HCWs to accept the COVID-19 vaccine. Prior studies also found that physicians were more likely than other HCWs to get COVID-19 vaccination (64, 65). It could be due to physicians having a better understanding of the coronavirus and its vaccine than the general public (66). Physicians may also have witnessed the disease's fatality, which may increase the likelihood that they will accept the COVID-19 vaccine.

The present study found that among unvaccinated HCWs, being 18–29 years old and working for <5 years were common risk factors for all psychological outcomes except anxiety and insomnia symptoms. These findings are expected. Because before the vaccination program worldwide there were many studies found that being 18-29 years old and have worked <5 years, HCWs were associated with higher psychological outcomes during the SARS outbreak (59), Avian influenza A (H7N9) virus outbreak (67), and COVID-19 epidemic (68). Moreover, Mohammed et al. (69) showed that in a survey of 614 Ethiopian healthcare practitioners, participants under the age of 30 were nearly five times more likely to be hesitant of being vaccinated than those over the age of 40. Furthermore, a Turkish study of 212 research assistants and 23 specialty physicians at Akdeniz University Hospital found that physicians who had worked for <5 years had lower vaccine uptake (70). These findings could be explained by the lack of a clinical study for any immunization and no evidence for reference about the COVID-19 vaccine's safety in Bangladesh. According to Mahmud et al. (71), 64.86% of people postpone immunization until the vaccine's efficacy and safety are established, or COVID-19 becomes more deadly in Bangladesh. It could be one of the reasons for vaccine apprehension, particularly among the young and those with little work experience. False rumors and misconceptions concerning the COVID-19 vaccines must be dispelled, and individuals must be educated to the true scientific facts to boost vaccine acceptability among the younger generation and those with minimal job experience.

The present study demonstrated that participants who worked with a nurse were significantly more likely to suffer from symptoms of depression, anxiety, and stress among unvaccinated HCWs. This finding was supported by many other studies (72, 73). A systematic review of 33,062 HCWs, Pappa et al. (74) discovered that nurses have higher rates of psychological symptoms than other medical staff. It may be a fact that nurses are in charge of dealing with patients, performing more invasive procedures, and working for extended periods. This result also corresponds to other studies, which found that nurses were less likely than different working positions to be vaccinated (64, 65). According to Browne et al. (75), the prime causes for vaccine hesitancy among nurses were concerns about adverse effects, the novelty of the vaccine, and a lack of vaccine knowledge. To ensure the success of the national vaccination drive, tailored strategies and vaccine promotion campaigns aimed at nurses are required.

It was not surprising that respondents who worked as frontline workers and provided direct care to infected patients were a significantly higher chance of experiencing all psychological outcomes except anxiety and stress symptoms among unvaccinated HCWs. Many studies evaluated the traumatic effects of COVID-19 and revealed that frontline workers were reported higher symptoms of psychological consequences (68, 76). It could be due to a lack of antiviral materials, unpleasant feelings from patients, quarantine, and loss of communication with their families, all of which led to the poor psychological outcomes of frontline employees. Moreover, this conclusion contradicts recent studies (77) but it was aligned with Nguyen et al. (78), who reported a higher than anticipated rate of vaccine hesitancy among frontline HCWs. Furthermore, direct treatment to infected individuals was also connected to more unfavorable psychological outcomes during the SARS outbreak (5, 79), and the COVID-19 outbreak (76, 80). However, in a survey of 5,287 US healthcare workers, Shaw et al. (81) discovered that direct care providers and COVID-19 patient care providers had lower vaccine acceptability than others. They might want to hold off on analyzing more data until they can see how the vaccination impacts others and learn more about vaccine safety and effectiveness (64, 81). They are trusted and respected community members on public health issues. Their early-stage public acceptance and uptake of COVID-19 immunizations have the potential to affect public perceptions toward the vaccine. As a result, the COVID-19 vaccination should be accepted as soon as possible.

The present study suggests that respondents who were infected with COVID-19 had a significantly less chance of experiencing symptoms of general health problems, depression, and anxiety among unvaccinated HCWs. In contrast to our findings, a recent study done in Bangladesh by Rahman et al. (82) discovered that having positive COVID-19 test results were linked to higher psychological distress. Another study involving 283 HCWs in Saudi Arabia found that being positive for COVID-19 was not associated with an increase in depression and anxiety symptoms (83). However, our findings were consistent with a prior study involving 475 emergency HCWs in the United States, which discovered that those with a history of COVID-19 infection had lower vaccine intent (84). It could be because HCWs believe that natural infection has provided them with sufficient immunological protection against COVID-19, and thus vaccination will be ineffective. It is likely to be true in the short term. However, the risk of infection may increase with time since infection, given evidence concerning waning humoral immunity to COVID-19 and the short-lived immunity after infection with other coronaviruses (85). As a result, our novel findings could be beneficial to HCWs in those regards. However, this does not imply that they were knowingly infected with COVID-19. Whether or not they are infected, the current study suggests that they get vaccinated as soon as possible.

## Strengths and Limitations

The following are some of the study's advantages: first, the first nationwide study in Bangladesh that has evaluated the factors associated with psychological outcomes among vaccinated and unvaccinated HCWs against COVID-19 infection. Second, this research discovered that fully vaccinated HCWs against COVID-19 infection had a significant positive impact on their mental health. Third, this study had a large sample size and included a variety of HCWs, allowing meaningful findings to be drawn. Finally, this research will add to our understanding of SARS-CoV-2 vaccination and mental health, as well as assist governments and policymakers in developing an effective vaccine campaign to achieve vaccination coverage and herd immunity among HCWs and the general public during the SARS-CoV-2 outbreak.

This study provides novel findings on psychological outcomes and associated factors among vaccinated and unvaccinated Bangladeshi HCWs against COVID-19 infection, but its limitations must not be overlooked. First, psychological outcomes were determined using a self-report tool and an online survey. Future research should include clinical interviews or qualitative studies to get a more complete picture of the problem. Second, this online survey used convenience and snowball sampling, excluding HCWs who do not have internet access. Although the findings of this study may not be representative of all Bangladeshi HCWs, this should not have influenced our conclusions about the risk factors. Third, it is impossible to estimate the response rate because it is unclear how many people received the survey link. Finally, this study did not consider influencing factors such as which developer's vaccine you received and taking any vaccine after the age of 18.

## Conclusion

A lower prevalence of psychological outcomes was found among vaccinated HCWs against COVID-19 infection as well as risk factors for developing them. To control the infection and improve psychological outcomes, this study suggests emphasizing the vaccinated to unvaccinated HCWs as soon as possible. They also required special attention, health-related education, and psychological support.

## Data Availability Statement

The original contributions presented in the study are included in the article/Supplementary Materials, further inquiries can be directed to the corresponding author/s.

## Ethics Statement

The studies involving human participants were reviewed and approved by Institutional Ethical Review Board (IERB) of the Holy Family Red Crescent Medical College and Hospital, Dhaka, Bangladesh (Approval No: IERB/36) and the Ethics Committee of the First Affiliated Hospital, Zhejiang University School of Medicine. The patients/participants provided their written informed consent to participate in this study.

## Author Contributions

MA: conceptualization, methodology, formal analysis, and writing—original draft. MA, SP, and MM: data collection. MA, SP, MM, LN, and YX: writing—review, and editing. All authors contributed to the article and approved the submitted version.

## Funding

This study was supported by the National Natural Science Foundation of China (Grant Nos. 81801340 and 81971271) and the Natural Science Foundation of Zhejiang Province (Grant No. LQ18H090001). The funding sources had no involvement in the study design, data collection, analysis, interpretation of data, writing of the report, or the decision to submit the paper for publication.

## Conflict of Interest

The authors declare that the research was conducted in the absence of any commercial or financial relationships that could be construed as a potential conflict of interest.

## Publisher's Note

All claims expressed in this article are solely those of the authors and do not necessarily represent those of their affiliated organizations, or those of the publisher, the editors and the reviewers. Any product that may be evaluated in this article, or claim that may be made by its manufacturer, is not guaranteed or endorsed by the publisher.

## References

[1] World Health Organization. WHO Coronavirus (COVID-19) Dashboard (2021). Available online at: https://covid19.who.int/ (accessed September 7, 2021).

[2] Kang L Li Y Hu S Chen M Yang C Yang BX. The mental health of medical workers in Wuhan, China dealing with the 2019 novel coronavirus. Lancet Psychiatry. (2020) 7:e14. 10.1016/S2215-0366(20)30047-X 32035030 PMC7129673

[3] Chong MY Wang WC Hsieh WC Lee CY Chiu NM Yeh WC. Psychological impact of severe acute respiratory syndrome on health workers in a tertiary hospital. Br J Psychiatry. (2004) 185:127–33. 10.1192/bjp.185.2.127 15286063

[4] Maunder R. The experience of the 2003 SARS outbreak as a traumatic stress among frontline healthcare workers in Toronto: lessons learned. Philos Trans R Soc Lond B Biol Sci. (2004) 359:1117–25. 10.1098/rstb.2004.1483 15306398 PMC1693388

[5] Maunder RG Lancee WJ Balderson KE Bennett JP Borgundvaag B Evans S. Long-term psychological and occupational effects of providing hospital healthcare during SARS outbreak. Emerg Infect Dis. (2006) 12:1924–32. 10.3201/eid1212.060584 17326946 PMC3291360

[6] McAlonan GM Lee AM Cheung V Cheung C Tsang KW Sham PC. Immediate and sustained psychological impact of an emerging infectious disease outbreak on health care workers. Can J Psychiatry. (2007) 52:241–7. 10.1177/070674370705200406 17500305

[7] Bruffaerts R Voorspoels W Jansen L Kessler RC Mortier P Vilagut G. Suicidality among healthcare professionals during the first COVID19 wave. J Affect Disord. (2021) 283:66–70. 10.1016/j.jad.2021.01.013 33524660 PMC7832920

[8] Sanghera J Pattani N Hashmi Y Varley KF Cheruvu MS Bradley A. The impact of SARS-CoV-2 on the mental health of healthcare workers in a hospital setting-a systematic review. J Occup Health. (2020) 62:e12175. 10.1002/1348-9585.12175 33131192 PMC7603426

[9] Salazar de Pablo G Vaquerizo-Serrano J Catalan A Arango C Moreno C Ferre F. Impact of coronavirus syndromes on physical and mental health of health care workers: Systematic review and meta-analysis. J Affect Disord. (2020) 275:48–57. 10.1016/j.jad.2020.06.022 32658823 PMC7314697

[10] Tasnim R Sujan MSH Islam MS Ritu AH Siddique MAB Toma TY. Prevalence and correlates of anxiety and depression in frontline healthcare workers treating people with COVID-19 in Bangladesh. BMC Psychiatry. (2021) 21:271. 10.1186/s12888-021-03243-w 34034679 PMC8146174

[11] Institute of Epidemiology, Disease Control and Research (IEDCR). Covid-19 Status Bangladesh (2020). Available online at: https://iedcr.gov.bd/covid-19/ (accessed September 13, 2021).

[12] Directorate General of Health Services. Novel Coronavirus (COVID-19) Press Release (2021). Available online at: http://103.247.238.92/webportal/pages/covid19-vaccination-update.php (accessed September 12, 2021).

[13] Bangladesh Medical Association. District Wise Total Number of Affected Doctor, Nurse & Staff (COVID 19+) (2021). Available online at: https://bma.org.bd/ (accessed October 8, 2021).

[14] Bangladesh Medical Association. List of Death Doctors Due to COVID-19 (2021). Available online at: https://bma.org.bd/ (accessed October 8, 2021).

[15] Repon MAU Pakhe SA Quaiyum S Das R Daria S Islam MR. Effect of COVID-19 pandemic on mental health among Bangladeshi healthcare professionals: a cross-sectional study. Sci Prog. (2021) 104:368504211026409. 10.1177/00368504211026409 34166132 PMC10455000

[16] Wang J Peng Y Xu H Cui Z Williams RO 3rd. The COVID-19 vaccine race: challenges and opportunities in vaccine formulation. AAPS PharmSciTech. (2020) 21:225. 10.1208/s12249-020-01744-7 32761294 PMC7405756

[17] Murphy J Vallières F Bentall RP Shevlin M McBride O Hartman TK. Psychological characteristics associated with COVID-19 vaccine hesitancy and resistance in Ireland and the United Kingdom. Nat Commun. (2021) 12:29. 10.1038/s41467-020-20226-9 33397962 PMC7782692

[18] McGill COVID19 Vaccine Tracker Team 2021. COVID-19 Vaccine Tracker (2021). Available online at: https://covid19.trackvaccines.org/agency/who/ (accessed September 22, 2021).

[19] Bangladesh Sangbad Sangstha. The Vaccine Has Been Administered to 1 Crore 4 Lakh 13 Thousand 606 People in the Country (2021). Available online at: https://www.bssnews.net/bangla/news-flash/3000 (accessed September 9, 2021).

[20] Sanche S Lin YT Xu C Romero-Severson E Hengartner N Ke R. High contagiousness and rapid spread of severe acute respiratory syndrome coronavirus 2. Emerg Infect Dis. (2020) 26:1470–7. 10.3201/eid2607.200282 32255761 PMC7323562

[21] Vahratian A Blumberg SJ Terlizzi EP Schiller JS. Symptoms of anxiety or depressive disorder and use of mental health care among adults during the COVID-19 pandemic - United States, August 2020-February 2021. MMWR Morb Mortal Wkly Rep. (2021) 70:490–4. 10.15585/mmwr.mm7013e2 33793459 PMC8022876

[22] Bendau A Plag J Petzold MB Ströhle A. COVID-19 vaccine hesitancy and related fears and anxiety. Int Immunopharmacol. (2021) 97:107724. 10.1016/j.intimp.2021.107724 33951558 PMC8078903

[23] Zheng YB Sun J Liu L Zhao YM Yan W Yuan K. COVID-19 vaccine-related psychological stress among general public in China. Front Psychiatry. (2021) 12:774504. 10.3389/fpsyt.2021.774504 34950070 PMC8689133

[24] Ilhan B Küpeli I. Secondary traumatic stress, anxiety, and depression among emergency healthcare workers in the middle of the COVID-19 outbreak: a cross-sectional study. Am J Emerg Med. (2022) 52:99–104. 10.1016/j.ajem.2021.11.051 34894474 PMC8641431

[25] Mahmud I Azad KAK Al Mamun A Hoque MM Mallik MU Moniruzzaman M. Psychological assessment of doctors working in a pandemic condition in Dhaka Medical College Hospital. J Bangladesh Coll Phys Surg. (2020) 2020:50–5. 10.3329/jbcps.v38i0.47446

[26] Goldberg DP Gater R Sartorius N Ustun TB Piccinelli M Gureje O. The validity of two versions of the GHQ in the WHO study of mental illness in general health care. Psychol Med. (1997) 27:191–7. 10.1017/S0033291796004242 9122299

[27] Islam MN Iqbal K. Mental health and social support. Chittagong Univ J Biol Sci. (2008) 3:95–107. 10.3329/cujbs.v3i1.13410

[28] Kroenke K Spitzer RL Williams JB. The Patient Health Questionnaire-2: validity of a two-item depression screener. Med Care. (2003) 2003:1284–92. 10.1097/01.MLR.0000093487.78664.3C 14583691

[29] Chowdhury AN Ghosh S Sanyal D. Bengali adaptation of brief patient health questionnaire for screening depression at primary care. J Indian Med Assoc. (2004) 102:544–7. Available online at: https://www.researchgate.net/publication/7853095_Bengali_adaptation_of_Brief_Patient_Health_Questionnaire_for_screening_depression_at_primary_care (accessed September 26, 2021). 15887819

[30] Bhattacharjee A Bansal V Juman MKI. COVID-19 emergency: faux healthcare service causes distress and life dissatisfaction 2. AJMAH. (2021) 18:53–61. 10.9734/ajmah/2020/v18i1230290

[31] Kroenke K Spitzer RL Williams JB Monahan PO Löwe B. Anxiety disorders in primary care: prevalence, impairment, comorbidity, and detection. Ann Intern Med. (2007) 146:317–25. 10.7326/0003-4819-146-5-200703060-00004 17339617

[32] Haque M Das C Ara R Alam M Ullah S Hossain Z. Prevalence of generalized anxiety disorder and its effect on daily living in the rural community of Rajshahi. TAJ J Teach Assoc. (2014) 27:14–23. 10.3329/taj.v27i1.37603

[33] Cohen S Kamarck T Mermelstein R. A global measure of perceived stress. J Health Soc Behav. (1983) 24:385–96. 10.2307/2136404 6668417

[34] Chakraborti A Ray P Sanyal D Thakurta RG Bhattacharayya AK Mallick AK. Assessing perceived stress in medical personnel: in search of an appropriate scale for the Bengali population. Indian J Psychol Med. (2013) 35:29–33. 10.4103/0253-7176.112197 23833339 PMC3701356

[35] Mozumder MK. Validation of Bengali perceived stress scale among LGBT population. BMC Psychiatry. (2017) 17:1–7. 10.1186/s12888-017-1482-0 28851332 PMC5576282

[36] Prins A Bovin MJ Smolenski DJ Marx BP Kimerling R Jenkins-Guarnieri MA. The primary care PTSD screen for DSM-5 (PC-PTSD-5): development and evaluation within a veteran primary care sample. J Gen Intern Med. (2016) 31:1206–11. 10.1007/s11606-016-3703-5 27170304 PMC5023594

[37] Ahmed S Ahsan MS Khan R Hasan M Ferdous F Shahjahan H. Psychological impact of COVID-19 pandemic on frontline health care workers in Bangladesh: a cross-sectional study. BJPsych Open. (2021) 7:S232-3. 10.1192/bjo.2021.621

[38] Bastien CH Vallières A Morin CM. Validation of the insomnia severity index as an outcome measure for insomnia research. Sleep Med. (2001) 2:297–307. 10.1016/S1389-9457(00)00065-4 11438246

[39] Mamun MA Alimoradi Z Gozal D Manzar MD Broström A Lin C-Y. Validating Insomnia Severity Index (ISI) in a Bangladeshi Population: using classical test theory and rasch analysis. Int J Environ Res Public Health. (2022) 19:225. 10.3390/ijerph19010225 35010485 PMC8750940

[40] Hughes ME Waite LJ Hawkley LC Cacioppo JT. A short scale for measuring loneliness in large surveys: results from two population-based studies. Res Aging. (2004) 26:655–72. 10.1177/0164027504268574 18504506 PMC2394670

[41] Ahmed O. Psychometric assessment of the Bangla UCLA loneliness scale-version 3. Bangladesh J Psychol. (2019) 22:35–53. Available online at: https://www.researchgate.net/publication/339434492_Psychometric_Assessment_of_the_Bangla_UCLA_Loneliness_Scale_-_version_3 (accessed September 28, 2021).

[42] Kocalevent R-D Berg L Beutel ME Hinz A Zenger M Härter M. Social support in the general population: standardization of the Oslo social support scale (OSSS-3). BMC Psychol. (2018) 6:1–8. 10.1186/s40359-018-0249-9 30016997 PMC6050647

[43] Beaton DE Bombardier C Guillemin F Ferraz MB. Guidelines for the process of cross-cultural adaptation of self-report measures. Spine. (2000) 25:3186–91. 10.1097/00007632-200012150-00014 11124735

[44] Haddaden M Aldabain L Patel N Maharaj A Saif A Imam Z. Health care workers attitudes toward COVID-19 vaccination and the effect on personal and professional life. J Community Hosp Intern Med Perspect. (2021) 11:585–9. 10.1080/20009666.2021.1951943 34567445 PMC8462893

[45] Yuan Y Deng Z Chen M Yin D Zheng J Liu Y. Changes in mental health and preventive behaviors before and after COVID-19 vaccination: a propensity score matching (PSM) study. Vaccines. (2021) 9:1044. 10.3390/vaccines9091044 34579281 PMC8473427

[46] Chen S Aruldass AR Cardinal RN. Mental health outcomes after SARS-CoV-2 vaccination in the United States: a national cross-sectional study. J Affect Disord. (2022) 298:396–9. 10.1016/j.jad.2021.10.134 34774648 PMC8580571

[47] Gazibara T Thygesen LC Algren MH Tolstrup JS. Human papillomavirus vaccination and physical and mental health complaints among female students in secondary education institutions in Denmark. J Gen Intern Med. (2020) 35:2647–54. 10.1007/s11606-020-05845-8 32342482 PMC7458962

[48] Hasan MT Hossain S Safa F Anjum A Khan AH Koly KN. Prevalence of anxiety and depressive symptoms among physicians during the COVID-19 pandemic in Bangladesh: a cross-sectional study. medRxiv [Preprint]. (2020). 10.1101/2020.12.08.20245829 36606239 PMC9253439

[49] Ali M Uddin Z Ahsan NF Haque MZ Bairagee M Khan SA. Prevalence of mental health symptoms and its effect on insomnia among healthcare workers who attended hospitals during COVID-19 pandemic: a survey in Dhaka city. Heliyon. (2021) 7:e06985. 10.1016/j.heliyon.2021.e06985 34027184 PMC8120937

[50] Beutel ME Hettich N Ernst M Schmutzer G Tibubos AN Braehler E. Mental health and loneliness in the German general population during the COVID-19 pandemic compared to a representative pre-pandemic assessment. Sci Rep. (2021) 11:14946. 10.1038/s41598-021-94434-8 34294816 PMC8298418

[51] Malik A Malik J Ishaq U. Acceptance of COVID-19 vaccine in Pakistan among health care workers. PLoS ONE. (2021) 16:e0257237. 10.1371/journal.pone.0257237 34525110 PMC8443053

[52] Kuter BJ Browne S Momplaisir FM Feemster KA Shen AK Green-McKenzie J. Perspectives on the receipt of a COVID-19 vaccine: a survey of employees in two large hospitals in Philadelphia. Vaccine. (2021) 39:1693–700. 10.1016/j.vaccine.2021.02.029 33632563 PMC7885691

[53] Khatun MF Parvin MF Rashid MM Alam MS Kamrunnahar M Talukder A. Mental health of physicians during COVID-19 outbreak in Bangladesh: a web-based cross-sectional survey. Front Public Health. (2021) 9:592058. 10.3389/fpubh.2021.592058 33634065 PMC7902057

[54] Siddique AB Nath SD Islam MS Khan TH Pardhan S Amin MZ. Financial difficulties correlate with mental health among Bangladeshi residents amid COVID-19 pandemic: findings from a cross-sectional survey. Front Psychiatry. (2021) 12:755357. 10.3389/fpsyt.2021.755357 34955916 PMC8692668

[55] Abedin M Islam MA Rahman FN Reza HM Hossain MZ Hossain MA. Willingness to vaccinate against COVID-19 among Bangladeshi adults: understanding the strategies to optimize vaccination coverage. PLoS ONE. (2021) 16:e0250495. 10.1371/journal.pone.0250495 33905442 PMC8078802

[56] Banik R Islam MS Pranta MUR Rahman QM Rahman M Pardhan S. Understanding the determinants of COVID-19 vaccination intention and willingness to pay: findings from a population-based survey in Bangladesh. BMC Infect Dis. (2021) 21:892. 10.1186/s12879-021-06406-y 34465297 PMC8406014

[57] Parvej MI Sultana S Tabassum M Mannan SE Ahmed F. Determinants of COVID-19 vaccine acceptance and encountered side-effects among the vaccinated in Bangladesh. Asian Pac J Trop Med. (2021) 14:341. 10.4103/1995-7645.321610

[58] Ministry of Health & Family Welfare. Bangladesh Preparedness and Response Plan for COVID-19 (2020). Available online at: http://www.mohfw.gov.bd/index.php?option=com_docman&task=doc_download&gid=23359&lang=en (accessed September 21, 2021).

[59] Sim K Chong PN Chan YH Soon WS. Severe acute respiratory syndrome-related psychiatric and posttraumatic morbidities and coping responses in medical staff within a primary health care setting in Singapore. J Clin Psychiatry. (2004) 65:1120–7. 10.4088/JCP.v65n0815 15323599

[60] Li Z Ge J Yang M Feng J Qiao M Jiang R. Vicarious traumatization in the general public, members, and non-members of medical teams aiding in COVID-19 control. Brain Behav Immun. (2020) 88:916–9. 10.1016/j.bbi.2020.03.007 32169498 PMC7102670

[61] Chan AO Huak CY. Psychological impact of the 2003 severe acute respiratory syndrome outbreak on health care workers in a medium size regional general hospital in Singapore. Occup Med. (2004) 54:190–6. 10.1093/occmed/kqh027 15133143 PMC7107861

[62] Mosolova E Sosin D Mosolov S. Stress, anxiety, depression and burnout in frontline healthcare workers during two peaks of COVID-19 pandemic in Russia. Psychiatry Res. (2021) 306:114226. 10.1016/j.psychres.2021.114226 34619519 PMC8480133

[63] Angelo AT Alemayehu DS Dachew AM. Health care workers intention to accept COVID-19 vaccine and associated factors in southwestern Ethiopia, 2021. PLoS ONE. (2021) 16:e0257109. 10.1371/journal.pone.0257109 34478470 PMC8415602

[64] Gadoth A Halbrook M Martin-Blais R Gray A Tobin NH Ferbas KG. Cross-sectional assessment of COVID-19 vaccine acceptance among health care workers in Los Angeles. Ann Intern Med. (2021) 174:882–5. 10.7326/M20-7580 33556267 PMC7926184

[65] Gagneux-Brunon A Detoc M Bruel S Tardy B Rozaire O Frappe P. Intention to get vaccinations against COVID-19 in French healthcare workers during the first pandemic wave: a cross-sectional survey. J Hosp Infect. (2021) 108:168–73. 10.1016/j.jhin.2020.11.020 33259883 PMC7699157

[66] Lake EA Demissie BW Gebeyehu NA Wassie AY Gelaw KA Azeze GA. Knowledge, attitude and practice towards COVID-19 among health professionals in Ethiopia: a systematic review and meta-analysis. PLoS ONE. (2021) 16:e0247204. 10.1371/journal.pone.0247204 33606744 PMC7894858

[67] Tang L Pan L Yuan L Zha L. Prevalence and related factors of post-traumatic stress disorder among medical staff members exposed to H7N9 patients. Int J Nurs Sci. (2017) 4:63–7. 10.1016/j.ijnss.2016.12.002 31406720 PMC6626070

[68] Elbay RY Kurtulmuş A Arpacioglu S Karadere E. Depression, anxiety, stress levels of physicians and associated factors in Covid-19 pandemics. Psychiatry Res. (2020) 290:113130. 10.1016/j.psychres.2020.113130 32497969 PMC7255248

[69] Mohammed R Nguse TM Habte BM Fentie AM Gebretekle GB. COVID-19 vaccine hesitancy among Ethiopian healthcare workers. PLoS ONE. (2021) 16:e0261125. 10.1371/journal.pone.0261125 34919597 PMC8682893

[70] Polat HH Yalçin AN Öncel S. Influenza vaccination; Rates, knowledge and the attitudes of physicians in a university hospital. Turkiye Klin J Med Sci. (2010) 30:48–53. 10.5336/medsci.2008-8117

[71] Mahmud S Mohsin M Khan IA Mian AU Zaman MA. Knowledge, beliefs, attitudes and perceived risk about COVID-19 vaccine and determinants of COVID-19 vaccine acceptance in Bangladesh. PLoS ONE. (2021) 16:e0257096. 10.1371/journal.pone.0257096 34499673 PMC8428569

[72] Lai J Ma S Wang Y Cai Z Hu J Wei N. Factors associated with mental health outcomes among health care workers exposed to coronavirus disease 2019. JAMA Netw Open. (2020) 3:e203976. 10.1001/jamanetworkopen.2020.3976 32202646 PMC7090843

[73] Alenazi TH BinDhim NF Alenazi MH Tamim H Almagrabi RS Aljohani SM. Prevalence and predictors of anxiety among healthcare workers in Saudi Arabia during the COVID-19 pandemic. J Infect Public Health. (2020) 13:1645–51. 10.1016/j.jiph.2020.09.001 33032969 PMC7535800

[74] Pappa S Ntella V Giannakas T Giannakoulis VG Papoutsi E Katsaounou P. Prevalence of depression, anxiety, and insomnia among healthcare workers during the COVID-19 pandemic: a systematic review and meta-analysis. Brain Behav Immun. (2020) 88:901–7. 10.1016/j.bbi.2020.05.026 32437915 PMC7206431

[75] Browne SK Feemster KA Shen AK Green-McKenzie J Momplaisir FM Faig W. Coronavirus disease 2019 (COVID-19) vaccine hesitancy among physicians, physician assistants, nurse practitioners, and nurses in two academic hospitals in Philadelphia. Infect Control Hosp Epidemiol. (2021) 20:1–9. 10.1017/ice.2021.410 34538290 PMC8503076

[76] Ceri V Cicek I. Psychological well-being, depression and stress during COVID-19 pandemic in Turkey: a comparative study of healthcare professionals and non-healthcare professionals. Psychol Health Med. (2021) 26:85–97. 10.1080/13548506.2020.1859566 33320723

[77] Kumar R Beniwal K Bahurupi Y Kant R Bairwa M. Determinants of COVID-19 vaccination willingness among health care workers: a quick online survey in India. Korean J Fam Med. (2021) 42:445–52. 10.4082/kjfm.21.0071 34871485 PMC8648495

[78] Nguyen LH Joshi AD Drew DA Merino J Ma W Lo CH. Racial and ethnic differences in COVID-19 vaccine hesitancy and uptake. medRxiv [Preprint]. (2021). 10.1101/2021.02.25.21252402 33655271 PMC7924296

[79] Li L Cheng S Gu J. SARS infection among health care workers in Beijing, China. JAMA. (2003) 290:2662–3. 10.1001/jama.290.20.2662 14645305

[80] Zubayer AA Rahman ME Islam MB Babu SZD Rahman QM Bhuiyan M. Psychological states of Bangladeshi people four months after the COVID-19 pandemic: an online survey. Heliyon. (2020) 6:e05057. 10.1016/j.heliyon.2020.e05057 33015396 PMC7521899

[81] Shaw J Stewart T Anderson KB Hanley S Thomas SJ Salmon DA. Assessment of US healthcare personnel attitudes towards Coronavirus Disease 2019 (COVID-19) vaccination in a large university healthcare system. Clin Infect Dis. (2021) 73:1776–83. 10.1093/cid/ciab054 33491049 PMC7929026

[82] Rahman MA Rahman S Wazib A Arafat SMY Chowdhury ZZ Uddin BMM. COVID-19 related psychological distress, fear and coping: identification of high-risk groups in Bangladesh. Front Psychiatry. (2021) 12:718654. 10.3389/fpsyt.2021.718654 34484005 PMC8414638

[83] Sultan S Bashar A Nomani I Tabassum A Iqbal MS Fallata EO. Impact of COVID-19 pandemic on psychological health of a sample of the health care workers in the western region of Kingdom of Saudi Arabia. Middle East Curr Psychiatry. (2022) 29:1–11. 10.1186/s43045-022-00174-4

[84] Pacella-LaBarbara ML Park YL Patterson PD Doshi A Guyette MK Wong AH. COVID-19 vaccine uptake and intent among emergency healthcare workers: a cross-sectional survey. J Occup Environ Med. (2021) 63:852–6. 10.1097/JOM.0000000000002298 34138823 PMC8478093

[85] Anderson RM Vegvari C Truscott J Collyer BS. Challenges in creating herd immunity to SARS-CoV-2 infection by mass vaccination. Lancet. (2020) 396:1614–6. 10.1016/S0140-6736(20)32318-7 33159850 PMC7836302

