# Supplementary material for: Factors Associated With Psychological Outcomes Among Vaccinated and Unvaccinated Health Care Workers Against COVID-19 Infection in Bangladesh
Source: Front Med (Lausanne). 2022 Mar 24;9:852922. doi: 10.3389/fmed.2022.852922 (PMC8988188; doi:10.3389/fmed.2022.852922)
Supplement: Supplementary file 1 [file Data_Sheet_1.docx]

**Supplement Figure S1.** District-wise total number of COVID-19 infected health care workers in Bangladesh from March 2020 to August 2021 (Data Source: Bangladesh Medical Association).

**Supplement Figure S2.** District-wise daily COVID-19 related deaths health care workers in Bangladesh from April 2020 to August 2021 (Data Source: Bangladesh Medical Association).

**Supplement Figure S3.** Daily COVID-19 vaccination in Bangladesh from January to August 2021 (Source: Directorate General of Health Services).
