# Supplementary material for: Factors Associated With Psychological Outcomes Among Vaccinated and Unvaccinated Health Care Workers Against COVID-19 Infection in Bangladesh
Source: Front Med (Lausanne). 2022 Mar 24;9:852922. doi: 10.3389/fmed.2022.852922 (PMC8988188; doi:10.3389/fmed.2022.852922)
Supplement: Supplementary file 2 [file Data_Sheet_2.docx]

**Supplement Table S1.** Univariate logistic regression analysis of factors associated with psychological outcomes among vaccinated and unvaccinated health care workers against COVID-19 infection.

| **Psychological outcomes** | **Sex** | | | | | |  | **Age, y** | | | | | | | |  | **Residence** | | | |  | **Marital status** | | | | | |  | | **Having children** | | | |
| --- | --- | --- | --- | --- | --- | --- | --- | --- | --- | --- | --- | --- | --- | --- | --- | --- | --- | --- | --- | --- | --- | --- | --- | --- | --- | --- | --- | --- | --- | --- | --- | --- | --- |
|  | **Male** | | **Female** | | **Not interested** | |  | **18-29** | | **30-39** | | **40-49** | | **≥50** | |  | **Urban** | | **Rural** | |  | **Single** | | **Married** | | **Divorced/separated/widowed** | |  | | **Yes** | | **No** | |
|  | **COR**  **(95% CI)** | ***p* value** | **COR**  **(95% CI)** | ***p* value** | **COR**  **(95% CI)** | ***p* value** |  | **COR**  **(95% CI)** | ***p***  **value** | **COR**  **(95% CI)** | ***p* value** | **COR**  **(95% CI)** | ***p* value** | **COR**  **(95% CI)** | ***p* value** |  | **COR**  **(95% CI)** | ***p* value** | **COR**  **(95% CI)** | ***p* value** |  | **COR**  **(95% CI)** | ***p* value** | **COR**  **(95% CI)** | ***p***  **value** | **COR**  **(95% CI)** | ***p* value** |  | **COR**  **(95% CI)** | | ***p* value** | **COR**  **(95% CI)** | ***p* value** |
| **General health problems** | | | | | | | | | | | | | | | | | | | | | | | | | | | | | | | | | |
| Total | 0.41  (0.22-0.75) | ≤0.01 | 0.45  (0.24-0.83) | ≤0.01 | 1  [Reference] |  |  | 3.55  (2.57-4.91) | ≤0.01 | 1.93  (1.39-2.69) | ≤0.01 | 1.72  (1.22-2.44) | ≤0.01 | 1  [Reference] |  |  | 2.20  (1.80-2.68) | ≤0.01 | 1  [Reference] |  |  | 2.47  (1.78-3.44) | ≤0.01 | 1.10  (0.82-1.48) | 0.51 | 1  [Reference] |  |  | 0.53  (0.44-0.64) | | ≤0.01 | 1  [Reference] |  |
| Vaccinated Health Care Workers | 0.63  (0.29-1.39) | ≤0.05 | 1.11  (0.50-2.46) | 0.79 | 1  [Reference] |  |  | 17.8  (10.0-31.8) | ≤0.01 | 6.11  (3.63-10.2) | ≤0.01 | 2.62  (1.61-4.28) | ≤0.01 | 1  [Reference] |  |  | 12.5  (8.50-18.6) | ≤0.01 | 1  [Reference] |  |  | 0.70  (0.20-2.43) | 0.58 | 0.14  (0.04-0.45) | ≤0.001 | 1  [Reference] |  |  | 0.20  (0.13-0.31) | | ≤0.01 | 1  [Reference] |  |
| Unvaccinated Health Care Workers | 2.29  (0.46-11.4) | 0.31 | 1.87  (0.37-9.36) | 0.44 | 1  [Reference] |  |  | 2.26  (1.36-3.75) | ≤0.001 | 1.42  (0.85-2.37) | 0.18 | 1.38  (0.80-2.39) | 0.24 | 1  [Reference] |  |  | 1.09  (0.83-1.45) | 0.51 | 1  [Reference] |  |  | 1.97  (1.28-3.04) | ≤0.01 | 1.34  (0.90-1.99) | 0.13 | 1  [Reference] |  |  | 0.68  (0.53-0.87) | | ≤0.01 | 1  [Reference] |  |
| **Depression symptoms** | | |  | | | | | | | | | | | |  | | | | | | | | | | | | | | | | | | |
| Total | 0.35  (0.16-0.79) | ≤0.01 | 0.40  (0.18-0.89) | ≤0.05 | 1  [Reference] |  |  | 2.85  (2.04-3.98) | ≤0.01 | 2.55  (1.81-3.60) | ≤0.01 | 2.00  (1.39-2.87) | ≤0.01 | 1  [Reference] |  |  | 1.50  (1.20-1.87) | ≤0.01 | 1  [Reference] |  |  | 2.18  (1.50-3.15) | ≤0.01 | 1.16  (0.84-1.61) | 0.35 | 1  [Reference] |  |  | 0.63  (0.52-0.78) | | ≤0.01 | 1  [Reference] |  |
| Vaccinated Health Care Workers | 0.43  (0.16-1.12) | 0.08 | 0.61  (0.23-1.61) | 0.32 | 1  [Reference] |  |  | 4.84  (2.95-7.94) | ≤0.01 | 5.21  (2.98-9.09) | ≤0.01 | 2.60  (1.54-4.39) | ≤0.01 | 1  [Reference] |  |  | 3.92  (2.74-5.60) | ≤0.01 | 1  [Reference] |  |  | 0.84  (0.34-2.10) | 0.72 | 0.39  (0.16-0.93) | ≤0.05 | 1  [Reference] |  |  | 0.44  (0.31-0.64) | | ≤0.01 | 1  [Reference] |  |
| Unvaccinated Health Care Workers | 0.71  (0.14-3.59) | 0.68 | 0.69  (0.13-3.49) | 0.66 | 1  [Reference] |  |  | 2.16  (1.34-3.48) | ≤0.001 | 2.03  (1.25-3.29) | ≤0.01 | 1.71  (1.02-2.87) | ≤0.05 | 1  [Reference] |  |  | 0.84  (0.62-1.13) | 0.26 | 1  [Reference] |  |  | 2.12  (1.36-3.31) | ≤0.001 | 1.38  (0.94-2.02) | 0.09 | 1  [Reference] |  |  | 0.76  (0.59-0.99) | | ≤0.05 | 1  [Reference] |  |
| **Anxiety symptoms** | | | | | | | | | | | | | | | | | | | | | | | | | | | | | | | | | |
| Total | 1.29  (0.74-2.22) | 0.36 | 1.40  (0.81-2.43) | 0.22 | 1  [Reference] |  |  | 1.35  (0.95-1.90) | 0.08 | 1.26  (0.88-1.80) | 0.19 | 1.37  (0.93-2.00) | 0.10 | 1  [Reference] |  |  | 1.25  (1.00-1.55) | ≤0.05 | 1  [Reference] |  |  | 1.33  (0.93-1.91) | 0.11 | 1.12  (0.81-1.57) | 0.47 | 1  [Reference] |  |  | 0.90  (0.73-1.10) | | 0.31 | 1  [Reference] |  |
| Vaccinated Health Care Workers | 1.43  (0.79-2.59) | 0.23 | 1.80  (0.99-3.27) | ≤0.05 | 1  [Reference] |  |  | 1.52  (0.95-2.43) | 0.08 | 1.15  (0.70-1.88) | 0.57 | 1.47  (0.87-2.49) | 0.14 | 1  [Reference] |  |  | 1.39  (1.02-1.91) | ≤0.05 | 1  [Reference] |  |  | 0.99  (0.52-1.85) | 0.97 | 0.77  (0.42-1.42) | 0.41 | 1  [Reference] |  |  | 0.83  (0.62-1.11) | | 0.22 | 1  [Reference] |  |
| Unvaccinated Health Care Workers | 0.41  (0.05-3.36) | 0.40 | 0.39  (0.04-3.23) | 0.38 | 1  [Reference] |  |  | 1.21  (0.72-2.01) | 0.46 | 1.35  (0.80-2.28) | 0.25 | 1.27  (0.72-2.22) | 0.40 | 1  [Reference] |  |  | 1.13  (0.83-1.53) | 0.43 | 1  [Reference] |  |  | 1.47  (0.93-2.33) | 0.64 | 1.35  (0.90-2.01) | 0.14 | 1  [Reference] |  |  | 0.97  (0.73-1.27) | | 0.83 | 1  [Reference] |  |
| **Stress symptoms** | | | | | | | | | | | | | | | | | | | | | | | |  | | | | | | | | | |
| Total | 1.56  (0.93-2.60) | 0.08 | 1.38  (0.83-2.30) | 0.21 | 1  [Reference] |  |  | 1.42  (1.02-1.96) | ≤0.05 | 1.11  (0.79-1.54) | 0.53 | 1.39  (0.98-1.99) | 0.06 | 1  [Reference] |  |  | 1.27  (1.04-1.56) | ≤0.01 | 1  [Reference] |  |  | 1.13  (0.81-1.59) | 0.44 | 0.94  (0.69-1.29) | 0.73 | 1  [Reference] |  |  | 0.88  (0.74-1.06) | | 0.20 | 1  [Reference] |  |
| Vaccinated Health Care Workers | 1.45  (0.82-2.56) | 0.19 | 1.22  (0.69-2.15) | 0.47 | 1  [Reference] |  |  | 1.14  (0.73-1.78) | 0.54 | 1.07  (0.67-1.71) | 0.76 | 1.13  (0.69-1.84) | 0.61 | 1  [Reference] |  |  | 1.50  (1.12-2.00) | ≤0.01 | 1  [Reference] |  |  | 1.02  (0.59-1.76) | 0.93 | 1.03  (0.61-1.74) | 0.90 | 1  [Reference] |  |  | 0.94  (0.73-1.23) | | 0.69 | 1  [Reference] |  |
| Unvaccinated Health Care Workers | 3.25  (0.76-13.7) | 0.10 | 2.99  (0.70-12.6) | 0.13 | 1  [Reference] |  |  | 1.81  (1.13-2.92) | ≤0.01 | 1.22  (0.76-1.97) | 0.40 | 1.78  (1.05-3.00) | ≤0.05 | 1  [Reference] |  |  | 1.09  (0.82-1.45) | 0.55 | 1  [Reference] |  |  | 1.33  (0.85-2.07) | 0.20 | 0.88  (0.60-1.30) | 0.54 | 1  [Reference] |  |  | 0.83  (0.65-1.07) | | 0.16 | 1  [Reference] |  |
| **Post-traumatic stress disorder symptoms** | | | | | | | | | | | | | | | | | | | | | | | | | | | | | | | | | |
| Total | 1.18  (0.67-2.08) | 0.54 | 0.97  (0.55-1.70) | 0.91 | 1  [Reference] |  |  | 0.15  (0.09-0.26) | ≤0.01 | 0.22  (0.13-0.39) | ≤0.01 | 0.63  (0.35-1.13) | 0.12 | 1  [Reference] |  |  | 1.03  (0.82-1.28) | 0.77 | 1  [Reference] |  |  | 0.17  (0.10-0.28) | ≤0.01 | 0.32  (0.19-0.52) | ≤0.01 | 1  [Reference] |  |  | 2.15  (1.76-2.63) | | ≤0.01 | 1  [Reference] |  |
| Vaccinated Health Care Workers | 1.05  (0.54-2.04) | 0.87 | 1.05  (0.54-2.02) | 0.88 | 1  [Reference] |  |  | 0.20  (0.10-0.41) | ≤0.01 | 0.43  (0.21-0.89) | ≤0.05 | 0.73  (0.33-1.59) | 0.43 | 1  [Reference] |  |  | 0.95  (0.68-1.34) | 0.80 | 1  [Reference] |  |  | 0.22  (0.10-0.51) | ≤0.01 | 0.56  (0.24-1.26) | 0.16 | 1  [Reference] |  |  | 2.47  (1.81-3.39) | | ≤0.01 | 1  [Reference] |  |
| Unvaccinated Health Care Workers | 4.53  (1.07-19.2) | ≤0.05 | 3.12  (0.73-13.2) | 0.12 | 1  [Reference] |  |  | 0.11  (0.04-0.26) | ≤0.01 | 0.14  (0.05-0.33) | ≤0.01 | 0.51  (0.20-1.29) | 0.15 | 1  [Reference] |  |  | 1.11  (0.83-1.49) | 0.47 | 1  [Reference] |  |  | 0.13  (0.07-0.25) | ≤0.01 | 0.22  (0.11-0.40) | ≤0.01 | 1  [Reference] |  |  | 1.99  (1.52-2.59) | | ≤0.01 | 1  [Reference] |  |
| **Insomnia symptoms** | | | | | | | | | | | | | | | | | | | | | | | | | | | | | | | | | |
| Total | 0.45  (0.26-0.81) | ≤0.01 | 0.39  (0.22-0.70) | ≤0.01 | 1  [Reference] |  |  | 1.15  (0.84-1.57) | 0.38 | 1.08  (0.78-1.49) | 0.64 | 1.11  (0.79-1.57) | 0.53 | 1  [Reference] |  |  | 1.35  (1.11-1.64) | ≤0.01 | 1  [Reference] |  |  | 1.46  (1.06-2.01) | ≤0.01 | 1.12  (0.83-1.51) | 0.43 | 1  [Reference] |  |  | 0.88  (0.74-1.05) | | 0.16 | 1  [Reference] |  |
| Vaccinated Health Care Workers | 0.86  (0.43-1.73) | 0.68 | 0.69  (0.34-1.38) | ≤0.01 | 1  [Reference] |  |  | 1.93  (1.22-3.05) | ≤0.01 | 1.94  (1.18-3.19) | ≤0.01 | 1.72  (1.03-2.87) | ≤0.05 | 1  [Reference] |  |  | 2.52  (1.84-3.43) | ≤0.01 | 1  [Reference] |  |  | 0.62  (0.30-1.28) | 0.19 | 0.48  (0.24-0.97) | ≤0.05 | 1  [Reference] |  |  | 0.73  (0.55-0.99) | | ≤0.05 | 1  [Reference] |  |
| Unvaccinated Health Care Workers | 1.81  (0.36-9.05) | 0.47 | 1.43  (0.28-7.17) | 0.66 | 1  [Reference] |  |  | 0.89  (0.54-1.45) | 0.64 | 1.01  (0.62-1.66) | 0.94 | 0.88  (0.52-1.50) | 0.64 | 1  [Reference] |  |  | 0.96  (0.72-1.28) | 0.79 | 1  [Reference] |  |  | 1.14  (0.74-1.78) | 0.53 | 1.12  (0.76-1.67) | 0.54 | 1  [Reference] |  |  | 1.00  (0.77-1.29) | | 0.98 | 1  [Reference] |  |
| **Loneliness symptoms** | | | | | | | | |  | | | | | | | | | | | | | | | | | | | | | | | | |
| Total | 1.85  (1.03-3.30) | ≤0.05 | 1.52  (0.85-2.71) | 0.15 | 1  [Reference] |  |  | 0.49  (0.30-0.80) | ≤0.01 | 0.41  (0.25-0.68) | ≤0.001 | 0.72  (0.42-1.24) | 0.24 | 1  [Reference] |  |  | 1.65  (1.30-2.10) | ≤0.01 | 1  [Reference] |  |  | 0.25  (0.13-0.47) | ≤0.01 | 0.26  (0.14-0.48) | ≤0.01 | 1  [Reference] |  |  | 1.20  (0.96-1.50) | | 0.10 | 1  [Reference] |  |
| Vaccinated Health Care Workers | 1.53  (0.77-3.04) | 0.22 | 2.05  (1.02-4.13) | ≤0.05 | 1  [Reference] |  |  | 1.70  (0.94-3.10) | 0.07 | 1.15  (0.62-2.12) | 0.64 | 0.90  (0.48-1.68) | 0.76 | 1  [Reference] |  |  | 3.81  (2.63-5.54) | ≤0.01 | 1  [Reference] |  |  | 0.74  (0.27-1.97) | 0.55 | 0.38  (0.15-0.97) | ≤0.05 | 1  [Reference] |  |  | 0.59  (0.41-0.86) | | ≤0.01 | 1  [Reference] |  |
| Unvaccinated Health Care Workers | 7.92  (1.86-33.7) | ≤0.01 | 4.69  (1.10-19.9) | ≤0.05 | 1  [Reference] |  |  | 0.06  (0.01-0.26) | ≤0.01 | 0.06  (0.01-0.26) | ≤0.01 | 0.23  (0.05-1.01) | ≤0.05 | 1  [Reference] |  |  | 0.91  (0.65-1.27) | 0.59 | 1  [Reference] |  |  | 0.11  (0.05-0.25) | ≤0.01 | 0.20  (0.09-0.45) | ≤0.01 | 1  [Reference] |  |  | 1.91  (1.42-2.58) | | ≤0.01 | 1  [Reference] |  |

| **Psychological outcomes** | **Highest education level** | | | | | | | |  | **Working position** | | | | | | | | | |  | **Work types** | | | |  | **Employment titles** | | | |
| --- | --- | --- | --- | --- | --- | --- | --- | --- | --- | --- | --- | --- | --- | --- | --- | --- | --- | --- | --- | --- | --- | --- | --- | --- | --- | --- | --- | --- | --- |
|  | **Bachelor (MBBS) or lower degree** | | **Post-graduate degree** | | **Doctoral degree** | | **Other** | |  | **Doctor** | | **Nurse** | | **Medical technician** | | **Hospital workers** | | **Other** | |  | **Frontline** | | **Second-line** | |  | **Senior** | | **Intermediate** | |
|  | **COR**  **(95% CI)** | ***p* value** | **COR**  **(95% CI)** | ***p* value** | **COR**  **(95% CI)** | ***p* value** | **COR**  **(95% CI)** | ***p* value** |  | **COR**  **(95% CI)** | ***p* value** | **COR**  **(95% CI)** | ***p* value** | **COR**  **(95% CI)** | ***p* value** | **COR**  **(95% CI)** | ***p* value** | **COR**  **(95% CI)** | ***p***  **value** |  | **COR**  **(95% CI)** | ***p***  **value** | **COR**  **(95% CI)** | ***p***  **value** |  | **COR**  **(95% CI)** | ***p***  **value** | **COR**  **(95% CI)** | ***p***  **value** |
| **General health problems** | | | | | | | | | | | | | | | | | | | | | | | | |  | | | | |
| Total | 8.29  (1.65-41.5) | ≤0.01 | 4.30  (0.86-21.4) | 0.07 | 2.83  (0.56-14.2) | 0.20 | 1  [Reference] |  |  | 0.66  (0.53-0.82) | ≤0.01 | 0.70  (0.49-0.99) | ≤0.05 | 0.73  (0.54-0.99) | ≤0.05 | 0.65  (0.49-0.86) | ≤0.01 | 1  [Reference] |  |  | 0.71  (0.59-0.85) | ≤0.01 | 1  [Reference] |  |  | 6.28  (0.79-49.7) | 0.08 | 12.9  (1.64-102.0) | ≤0.01 |
| Vaccinated Health Care Workers | 26.1  (4.55-150.1) | ≤0.01 | 12.0  (2.15-66.8) | ≤0.01 | 3.34  (0.59-18.7) | 0.17 | 1  [Reference] |  |  | 0.14  (0.08-0.24) | ≤0.01 | 0.46  (0.19-1.11) | 0.08 | 0.62  (0.25-1.56) | 0.31 | 0.69  (0.29-1.64) | 0.40 | 1  [Reference] |  |  | 0.69  (0.48-0.99) | ≤0.05 | 1  [Reference] |  |  | 7.02  (0.85-57.5) | 0.06 | 31.0  (3.77-255.1) | ≤0.001 |
| Unvaccinated Health Care Workers | - | - | - | - | - | - | 1  [Reference] |  |  | 0.68  (0.48-0.95) | ≤0.05 | 0.74  (0.46-1.19) | 0.21 | 1.08  (0.75-1.56) | 0.66 | 0.87  (0.62-1.24) | 0.46 | 1  [Reference] |  |  | 0.36  (0.28-0.46) | ≤0.01 | 1  [Reference] |  |  | - | - | - | - |
| **Depression symptoms** | | | | | | | | | | | | | | | | | | | | | | | | |  | | | | |
| Total | 6.60  (1.55-27.9) | ≤0.01 | 5.70  (1.35-24.0) | ≤0.01 | 3.28  (0.77-13.9) | 0.10 | 1  [Reference] |  |  | 0.88  (0.69-1.13) | 0.35 | 0.90  (0.60-1.34) | 0.60 | 1.24  (0.87-1.78) | 0.22 | 1.06  (0.77-1.46) | 0.71 | 1  [Reference] |  |  | 0.62  (0.50-0.76) | ≤0.01 | 1  [Reference] |  |  | 1.36  (0.40-4.56) | 0.61 | 2.29  (0.68-7.65) | 0.17 |
| Vaccinated Health Care Workers | 8.13  (1.58-41.7) | ≤0.01 | 6.35  (1.25-32.1) | ≤0.05 | 2.67  (0.52-13.6) | 0.23 | 1  [Reference] |  |  | 0.35  (0.23-0.55) | ≤0.01 | 0.93  (0.39-2.23) | 0.88 | 1.29  (0.51-3.21) | 0.58 | 0.64  (0.32-1.26) | 0.20 | 1  [Reference] |  |  | 0.78  (0.54-1.12) | 0.18 | 1  [Reference] |  |  | 1.25  (0.32-4.85) | 0.74 | 4.91  (1.24-19.4) | ≤0.01 |
| Unvaccinated Health Care Workers | - | - | - | - | - | - | 1  [Reference] |  |  | 1.22  (0.86-1.73) | 0.25 | 0.93  (0.58-1.51) | 0.79 | 1.63  (1.08-2.44) | ≤0.01 | 1.54  (1.06-2.25) | ≤0.05 | 1  [Reference] |  |  | 0.42  (0.32-0.54) | ≤0.01 | 1  [Reference] |  |  | 1.70  (0.10-27.8) | 0.70 | 1.78  (0.11-28.8) | 0.68 |
| **Anxiety symptoms** | | | | | | | | | | | | | | | | | | | | | | | | |  | | | | |
| Total | 1.04  (0.20-5.23) | 0.95 | 0.96  (0.19-4.79) | 0.96 | 0.90  (0.18-4.56) | 0.90 | 1  [Reference] |  |  | 0.64  (0.50-0.82) | ≤0.001 | 0.62  (0.42-0.91) | ≤0.01 | 0.72  (0.52-1.02) | 0.06 | 0.91  (0.65-1.26) | 0.57 | 1  [Reference] |  |  | 0.99  (0.81-1.22) | 0.99 | 1  [Reference] |  |  | 1.92  (0.57-6.45) | 0.29 | 2.68  (0.80-8.96) | 0.10 |
| Vaccinated Health Care Workers | 1.72  (0.31-9.59) | 0.53 | 1.60  (0.28-8.85) | 0.59 | 1.12  (0.20-6.31) | 0.89 | 1  [Reference] |  |  | 0.60  (0.42-0.84) | ≤0.01 | 0.92  (0.49-1.74) | 0.81 | 0.93  (0.51-1.71) | 0.83 | 0.95  (0.54-1.66) | 0.87 | 1  [Reference] |  |  | 0.80  (0.59-1.09) | 0.16 | 1  [Reference] |  |  | 2.00  (0.51-7.79) | 0.31 | 2.40  (0.62-9.28) | 0.20 |
| Unvaccinated Health Care Workers | - | - | - | - | - | - | 1  [Reference] |  |  | 0.70  (0.48-1.02) | 0.06 | 0.48  (0.29-0.79) | ≤0.01 | 0.66  (0.44-1.01) | ≤0.05 | 0.90  (0.60-1.36) | 0.63 | 1  [Reference] |  |  | 1.16  (0.88-1.54) | 0.26 | 1  [Reference] |  |  | 2.14  (0.13-34.9) | 0.59 | 3.40  (0.21-55.2) | 0.38 |
| **Stress symptoms** | | | | | | | | | | | | | | | | | | | | | | | | |  | | | | |
| Total | 1.15  (0.27-4.86) | 0.84 | 1.18  (0.28-4.99) | 0.81 | 0.93  (0.21-3.95) | 0.92 | 1  [Reference] |  |  | 1.09  (0.87-1.37) | 0.42 | 0.85  (0.59-1.21) | 0.38 | 1.15  (0.84-1.57) | 0.36 | 0.91  (0.69-1.21) | 0.55 | 1  [Reference] |  |  | 0.80  (0.66-0.96) | ≤0.05 | 1  [Reference] |  |  | 0.37  (0.07-1.75) | 0.21 | 0.35  (0.07-1.67) | 0.19 |
| Vaccinated Health Care Workers | 0.34  (0.04-2.98) | 0.33 | 0.42  (0.04-3.67) | 0.43 | 0.32  (0.03-2.85) | 0.31 | 1  [Reference] |  |  | 1.04  (0.76-1.41) | 0.79 | 1.25  (0.71-2.20) | 0.42 | 1.25  (0.73-2.14) | 0.39 | 0.84  (0.53-1.34) | 0.47 | 1  [Reference] |  |  | 0.77  (0.58-1.01) | 0.06 | 1  [Reference] |  |  | 0.54  (0.10-2.71) | 0.45 | 0.42  (0.08-2.07) | 0.28 |
| Unvaccinated Health Care Workers | - | - | - | - | - | - | 1  [Reference] |  |  | 1.21  (0.85-1.72) | 0.28 | 0.64  (0.40-1.03) | ≤0.05 | 1.10  (0.75-1.62) | ≤0.01 | 0.95  (0.66-1.36) | 0.79 | 1  [Reference] |  |  | 0.82  (0.63-1.05) | 0.12 | 1  [Reference] |  |  | - | - | - | - |
| **Post-traumatic stress disorder symptoms** | | | | | | | | | | | | | | |  |  | | | | | | | | |  | | | | |
| Total | - | - | - | - | - | - | 1  [Reference] |  |  | 0.98  (0.77-1.25) | 0.89 | 0.75  (0.52-1.09) | 0.13 | 1.01  (0.73-1.41) | 0.92 | 1.24  (0.90-1.70) | 0.18 | 1  [Reference] |  |  | 0.52  (0.42-0.64) | ≤0.01 | 1  [Reference] |  |  | 1.25  (0.15-10.2) | 0.83 | 0.34  (0.04-2.73) | 0.31 |
| Vaccinated Health Care Workers | - | - | - | - | - | - | 1  [Reference] |  |  | 1.00  (0.71-1.42) | 0.96 | 1.17  (0.61-2.23) | 0.62 | 1.17  (0.64-2.16) | 0.60 | 1.11  (0.64-1.92) | 0.71 | 1  [Reference] |  |  | 0.65  (0.47-0.91) | ≤0.01 | 1  [Reference] |  |  | 1.15  (0.13-9.90) | 0.89 | 0.76  (0.09-6.37) | 0.80 |
| Unvaccinated Health Care Workers | - | - | - | - | - | - | 1  [Reference] |  |  | 0.81  (0.57-1.15) | 0.24 | 0.59  (0.36-0.95) | 0.23 | 1.03  (0.69-1.54) | 0.85 | 1.40  (0.94-2.08) | 0.09 | 1  [Reference] |  |  | 0.38  (0.29-0.50) | ≤0.01 | 1  [Reference] |  |  | - | - | - | - |
| **Insomnia symptoms** | | | | | | | | | | | | | | | | | | | | | | | | |  | | | | |
| Total | 1.39  (0.34-5.62) | 0.64 | 1.14  (0.28-4.59) | 0.85 | 1.15  (0.28-4.67) | 0.84 | 1  [Reference] |  |  | 1.20  (0.96-1.49) | 0.09 | 0.55  (0.39-0.78) | ≤0.001 | 0.76  (0.56-1.01) | 0.06 | 0.80  (0.61-1.05) | 0.12 | 1  [Reference] |  |  | 0.72  (0.60-0.86) | ≤0.01 | 1  [Reference] |  |  | 0.76  (0.22-2.56) | 0.66 | 0.95  (0.28-3.15) | 0.93 |
| Vaccinated Health Care Workers | 3.39  (0.67-17.1) | 0.14 | 3.59  (0.71-18.0) | 0.12 | 2.42  (0.47-12.3) | 0.28 | 1  [Reference] |  |  | 0.64  (0.44-0.91) | ≤0.01 | 0.55  (0.30-1.00) | ≤0.05 | 0.41  (0.24-0.71) | ≤0.001 | 1.09  (0.60-1.98) | 0.76 | 1  [Reference] |  |  | 0.83  (0.61-1.14) | 0.26 | 1  [Reference] |  |  | 1.32  (0.34-5.13) | 0.68 | 2.92  (0.75-11.3) | 0.12 |
| Unvaccinated Health Care Workers | 0.56  (0.03-9.04) | 0.68 | 0.52  (0.03-8.43) | 0.64 | 0.55  (0.03-8.99) | 0.67 | 1  [Reference] |  |  | 1.15  (0.81-1.62) | 0.42 | 0.45  (0.25-0.79) | ≤0.01 | 1.42  (0.97-2.06) | 0.06 | 1.05  (0.73-1.51) | 0.77 | 1  [Reference] |  |  | 0.31  (0.24-0.41) | ≤0.01 | 1  [Reference] |  |  | 0.51  (0.03-8.30) | 0.63 | 0.53  (0.03-8.62) | 0.65 |
| **Loneliness symptoms** | | | | | | | | | | | | | | | | | | | | | | | | |  | | | | |
| Total | 1.41  (0.28-7.11) | 0.67 | 1.47  (0.29-7.38) | 0.63 | 1.86  (0.36-9.48) | 0.45 | 1  [Reference] |  |  | 1.09  (0.83-1.44) | 0.51 | 0.70  (0.47-1.06) | 0.09 | 1.61  (1.06-2.45) | ≤0.05 | 1.23  (0.86-1.77) | 0.25 | 1  [Reference] |  |  | 0.52  (0.41-0.67) | ≤0.01 | 1  [Reference] |  |  | 1.18  (0.24-5.66) | 0.82 | 1.21  (0.25-5.75) | 0.80 |
| Vaccinated Health Care Workers | 4.32  (0.76-24.4) | 0.09 | 2.94  (0.52-16.4) | 0.21 | 2.28  (0.40-12.9) | 0.35 | 1  [Reference] |  |  | 0.74  (0.48-1.12) | 0.15 | 0.88  (0.42-1.87) | 0.75 | 2.23  (0.85-5.84) | 0.10 | 1.50  (0.70-3.22) | 0.29 | 1  [Reference] |  |  | 0.51  (0.33-0.77) | ≤0.001 | 1  [Reference] |  |  | 0.90  (0.18-4.54) | 0.90 | 1.75  (0.34-8.88) | 0.49 |
| Unvaccinated Health Care Workers | - | - | - | - | - | - | 1  [Reference] |  |  | 1.34  (0.90-2.01) | 0.14 | 0.65  (0.39-1.07) | 0.09 | 1.72  (1.07-2.78) | ≤0.05 | 1.33  (0.87-2.03) | 0.17 | 1  [Reference] |  |  | 0.45  (0.33-0.61) | ≤0.01 | 1  [Reference] |  |  | - | - | - | - |

**Supplement Table S1.** Univariate logistic regression analysis of factors associated with psychological outcomes among vaccinated and unvaccinated health care workers against COVID-19 infection (continued).

| **Psychological outcomes** |  | | | | | |  | **Work experiences, y** | | | | | | | |  | **Socio economic status** | | | | | |  | **Living with family** | | | |  | **Smoking habit** | | | |
| --- | --- | --- | --- | --- | --- | --- | --- | --- | --- | --- | --- | --- | --- | --- | --- | --- | --- | --- | --- | --- | --- | --- | --- | --- | --- | --- | --- | --- | --- | --- | --- | --- |
|  | **Junior** | | **New** | | **Other** | |  | **≤5** | | **6-10** | | **11-19** | | **≥20** | |  | **Lower class** | | **Middle class** | | **Upper class** | |  | **Yes** | | **No** | |  | **Yes** | | **No** | |
|  | **COR**  **(95% CI)** | ***p* value** | **COR**  **(95% CI)** | ***p* value** | **COR**  **(95% CI)** | ***p* value** |  | **COR**  **(95% CI)** | ***p* value** | **COR**  **(95% CI)** | ***p* value** | **COR**  **(95% CI)** | ***p* value** | **COR**  **(95% CI)** | ***p* value** |  | **COR**  **(95% CI)** | ***p* value** | **COR**  **(95% CI)** | ***p***  **value** | **COR**  **(95% CI)** | ***p* value** |  | **COR**  **(95% CI)** | ***p* value** | **COR**  **(95% CI)** | ***p* value** |  | **COR**  **(95% CI)** | ***p* value** | **COR**  **(95% CI)** | ***p* value** |
| **General health problems** | | | | |  | | | | | | | | | | | | | | | | | | | | | | | | | | | |
| Total | 23.3  (2.97-183.3) | ≤0.01 | 20.7  (2.62-164.0) | ≤0.01 | 1  [Reference] |  |  | 3.13  (2.33-4.21) | ≤0.01 | 1.54  (1.11-2.14) | ≤0.01 | 1.32  (0.95-1.82) | 0.09 | 1  [Reference] |  |  | 2.40  (1.80-3.20) | ≤0.01 | 1.64  (1.26-2.12) | ≤0.01 | 1  [Reference] |  |  | 0.42  (0.35-0.51) | ≤0.01 | 1  [Reference] |  |  | 1.14  (0.93-1.38) | 0.18 | 1  [Reference] |  |
| Vaccinated Health Care Workers | 156.8  (18.8-1307.7) | ≤0.01 | 106.4  (12.0-937.4) | ≤0.01 | 1  [Reference] |  |  | 13.1  (7.96-21.7) | ≤0.01 | 4.42  (2.50-7.80) | ≤0.01 | 2.10  (1.31-3.35) | ≤0.01 | 1  [Reference] |  |  | 6.77  (3.47-13.2) | ≤0.01 | 1.46  (0.92-2.33) | 0.10 | 1  [Reference] |  |  | 0.14  (0.09-0.22) | ≤0.01 | 1  [Reference] |  |  | 1.60  (1.07-2.40) | ≤0.05 | 1  [Reference] |  |
| Unvaccinated Health Care Workers | - | - | - | - | 1  [Reference] |  |  | 2.20  (1.36-3.57) | ≤0.001 | 1.52  (0.90-2.55) | 0.11 | 1.31  (0.77-2.20) | 0.31 | 1  [Reference] |  |  | 1.62  (1.11-2.37) | ≤0.01 | 1.36  (0.96-1.93) | 0.08 | 1  [Reference] |  |  | 0.99  (0.76-1.30) | 0.97 | 1  [Reference] |  |  | 1.11  (0.85-1.85) | 0.42 | 1  [Reference] |  |
| **Depression symptoms** | | | | | | | | | | | | | | | | | | | | | | | | | | | | | | | | |
| Total | 3.49  (1.05-11.5) | ≤0.05 | 3.34  (0.99-11.2) | ≤0.05 | 1  [Reference] |  |  | 2.58  (1.89-3.52) | ≤0.01 | 2.14  (1.49-3.06) | ≤0.01 | 1.51  (1.07-2.12) | ≤0.01 | 1  [Reference] |  |  | 2.03  (1.47-2.80) | ≤0.01 | 1.42  (1.07-1.88) | ≤0.01 | 1  [Reference] |  |  | 0.53  (0.42-0.66) | ≤0.01 | 1  [Reference] |  |  | 1.22  (0.97-1.53) | 0.07 | 1  [Reference] |  |
| Vaccinated Health Care Workers | 7.26  (1.88-28.0) | ≤0.01 | 7.37  (1.77-30.6) | ≤0.01 | 1  [Reference] |  |  | 4.05  (2.55-6.41) | ≤0.01 | 4.32  (2.23-8.36) | ≤0.01 | 1.63  (0.99-2.68) | ≤0.05 | 1  [Reference] |  |  | 2.91  (1.58-5.36) | ≤0.001 | 1.28  (0.78-2.12) | 0.32 | 1  [Reference] |  |  | 0.45  (0.31-0.65) | ≤0.01 | 1  [Reference] |  |  | 1.44  (0.96-2.16) | 0.07 | 1  [Reference] |  |
| Unvaccinated Health Care Workers | 2.30  (0.14-37.1) | 0.55 | 2.78  (0.17-45.1) | 0.47 | 1  [Reference] |  |  | 2.21  (1.40-3.47) | ≤0.001 | 2.11  (1.29-3.45) | ≤0.01 | 1.70  (1.04-2.78) | ≤0.05 | 1  [Reference] |  |  | 1.56  (1.06-2.30) | ≤0.05 | 1.30  (0.92-1.84) | 0.13 | 1  [Reference] |  |  | 0.75  (0.56-1.00) | ≤0.05 | 1  [Reference] |  |  | 1.18  (0.89-1.57) | 0.23 | 1  [Reference] |  |
| **Anxiety symptoms** | | | | | | | | | | | | | | | | | | | | | | | | | | | | | | | | |
| Total | 2.55  (0.77-8.43) | 0.12 | 2.48  (0.74-8.32) | 0.14 | 1  [Reference] |  |  | 1.43  (1.04-1.97) | ≤0.05 | 1.20  (0.84-1.72) | 0.30 | 1.44  (1.01-2.07) | ≤0.05 | 1  [Reference] |  |  | 1.44  (1.05-1.97) | ≤0.05 | 1.34  (1.01-1.78) | ≤0.05 | 1  [Reference] |  |  | 0.88  (0.71-1.08) | 0.22 | 1  [Reference] |  |  | 0.88  (0.71-1.09) | 0.27 | 1  [Reference] |  |
| Vaccinated Health Care Workers | 2.84  (0.75-10.7) | 0.12 | 1.99  (0.50-7.78) | 0.32 | 1  [Reference] |  |  | 1.65  (1.08-2.50) | ≤0.01 | 1.05  (0.63-1.74) | 0.84 | 1.68  (1.01-2.77) | ≤0.05 | 1  [Reference] |  |  | 0.87  (0.51-1.48) | 0.62 | 0.80  (0.49-1.31) | 0.39 | 1  [Reference] |  |  | 1.02  (0.76-1.37) | 0.85 | 1  [Reference] |  |  | 1.06  (0.76-1.46) | 0.72 | 1  [Reference] |  |
| Unvaccinated Health Care Workers | 2.59  (0.16-41.8) | 0.50 | 3.37  (0.20-54.8) | 0.39 | 1  [Reference] |  |  | 1.22  (0.75-1.99) | 0.41 | 1.27  (0.75-2.15) | 0.37 | 1.25  (0.73-2.13) | 0.40 | 1  [Reference] |  |  | 1.88  (1.26-2.81) | ≤0.01 | 1.78  (1.25-2.55) | ≤0.001 | 1  [Reference] |  |  | 0.75  (0.55-1.02) | 0.07 | 1  [Reference] |  |  | 0.76  (0.57-1.02) | 0.07 | 1  [Reference] |  |
| **Stress symptoms** | | | |  | | | | | | | | | | | | | | | | | | | | | | | | | | | | |
| Total | 0.45  (0.09-2.10) | 0.31 | 0.44  (0.09-2.08) | 0.30 | 1  [Reference] |  |  | 1.36  (1.01-1.84) | ≤0.05 | 1.04  (0.75-1.46) | 0.77 | 1.24  (0.89-1.73) | 0.20 | 1  [Reference] |  |  | 1.03  (0.76-1.38) | 0.84 | 1.02  (0.78-1.34) | 0.84 | 1  [Reference] |  |  | 0.96  (0.80-1.16) | 0.73 | 1  [Reference] |  |  | 0.78  (0.64-0.95) | ≤0.01 | 1  [Reference] |  |
| Vaccinated Health Care Workers | 0.62  (0.12-3.06) | 0.56 | 0.44  (0.08-2.21) | 0.32 | 1  [Reference] |  |  | 1.11  (0.74-1.65) | 0.60 | 0.91  (0.55-1.48) | 0.70 | 1.04  (0.65-1.66) | 0.84 | 1  [Reference] |  |  | 0.85  (0.53-1.35) | 0.49 | 0.93  (0.60-1.44) | 0.75 | 1  [Reference] |  |  | 0.79  (0.61-1.04) | 0.09 | 1  [Reference] |  |  | 0.80  (0.60-1.07) | 0.14 | 1  [Reference] |  |
| Unvaccinated Health Care Workers | - | - | - | - | 1  [Reference] |  |  | 1.80  (1.14-2.83) | ≤0.01 | 1.30  (0.80-2.10) | 0.28 | 1.56  (0.96-2.55) | 0.07 | 1  [Reference] |  |  | 1.18  (0.80-1.73) | 0.38 | 1.07  (0.76-1.51) | 0.68 | 1  [Reference] |  |  | 1.19  (0.91-1.57) | 0.19 | 1  [Reference] |  |  | 0.77  (0.58-1.01) | ≤0.05 | 1  [Reference] |  |
| **Post-traumatic stress disorder symptoms** | | | | | | | | | | | | | | | | | | | | | | | | | | | | | | |  | |
| Total | 0.23  (0.03-1.82) | 0.16 | 0.14  (0.01-1.12) | 0.06 | 1  [Reference] |  |  | 0.15  (0.09-0.25) | ≤0.01 | 0.22  (0.12-0.37) | ≤0.01 | 0.35  (0.20-0.61) | ≤0.01 | 1  [Reference] |  |  | 0.87  (0.64-1.18) | 0.37 | 1.27  (0.95-1.70) | 0.09 | 1  [Reference] |  |  | 0.91  (0.74-1.11) | 0.37 | 1  [Reference] |  |  | 0.73  (0.59-0.90) | ≤0.01 | 1  [Reference] |  |
| Vaccinated Health Care Workers | 0.34  (0.04-2.81) | 0.32 | 0.23  (0.02-1.91) | 0.17 | 1  [Reference] |  |  | 0.26  (0.14-0.48) | ≤0.01 | 0.54  (0.27-1.10) | 0.09 | 0.83  (0.41-1.68) | 0.60 | 1  [Reference] |  |  | 0.92  (0.55-1.53) | 0.75 | 1.18  (0.73-1.92) | 0.49 | 1  [Reference] |  |  | 1.22  (0.90-1.66) | 0.18 | 1  [Reference] |  |  | 0.66  (0.48-0.91) | ≤0.01 | 1  [Reference] |  |
| Unvaccinated Health Care Workers | - | - | - | - | 1  [Reference] |  |  | 0.05  (0.01-0.16) | ≤0.01 | 0.07  (0.02-0.22) | ≤0.01 | 0.10  (0.03-0.35) | ≤0.01 | 1  [Reference] |  |  | 0.77  (0.52-1.13) | 0.18 | 1.24  (0.86-1.78) | 0.24 | 1  [Reference] |  |  | 0.81  (0.60-1.08) | 0.16 | 1  [Reference] |  |  | 0.79  (0.60-1.04) | 0.10 | 1  [Reference] |  |
| **Insomnia symptoms** | | | | | | | | | | | | | | | | | | | | | | | | | | | | | | | | |
| Total | 1.17  (0.35-3.87) | 0.79 | 0.98  (0.29-3.27) | 0.97 | 1  [Reference] |  |  | 1.09  (0.81-1.46) | 0.54 | 0.84  (0.60-1.16) | 0.29 | 0.84  (0.60-1.16) | 0.29 | 1  [Reference] |  |  | 1.42  (1.07-1.88) | ≤0.01 | 1.29  (1.00-1.67) | ≤0.05 | 1  [Reference] |  |  | 0.56  (0.46-0.67) | ≤0.01 | 1  [Reference] |  |  | 1.17  (0.96-1.41) | 0.10 | 1  [Reference] |  |
| Vaccinated Health Care Workers | 3.03  (0.79-11.4) | 0.10 | 3.14  (0.79-12.4) | 0.10 | 1  [Reference] |  |  | 1.63  (1.06-2.49) | ≤0.05 | 1.12  (0.66-1.89) | 0.66 | 1.26  (0.77-2.08) | 0.34 | 1  [Reference] |  |  | 1.42  (0.83-2.43) | 0.19 | 0.84  (0.52-1.36) | 0.50 | 1  [Reference] |  |  | 0.67  (0.50-0.91) | ≤0.01 | 1  [Reference] |  |  | 1.38  (0.98-1.93) | 0.06 | 1  [Reference] |  |
| Unvaccinated Health Care Workers | 0.52  (0.03-8.38) | 0.64 | 0.60  (0.03-9.79) | 0.72 | 1  [Reference] |  |  | 1.01  (0.63-1.61) | 0.96 | 1.20  (0.73-1.98) | 0.45 | 0.91  (0.54-1.51) | 0.71 | 1  [Reference] |  |  | 1.09  (0.74-1.61) | 0.64 | 1.22  (0.85-1.73) | 0.26 | 1  [Reference] |  |  | 0.77  (0.59-1.02) | 0.07 | 1  [Reference] |  |  | 1.21  (0.92-1.59) | 0.15 | 1  [Reference] |  |
| **Loneliness symptoms** | | | | | | | | | | | | | | | | | | | | | | | | | | | | | | | | |
| Total | 1.07  (0.23-5.03) | 0.92 | 0.65  (0.13-3.07) | 0.58 | 1  [Reference] |  |  | 0.57  (0.37-0.88) | ≤0.01 | 0.51  (0.32-0.83) | ≤0.01 | 0.67  (0.42-1.09) | 0.11 | 1  [Reference] |  |  | 1.10  (0.76-1.58) | 0.59 | 1.03  (0.74-1.43) | 0.84 | 1  [Reference] |  |  | 0.61  (0.48-0.78) | ≤0.01 | 1  [Reference] |  |  | 1.03  (0.81-1.33) | 0.76 | 1  [Reference] |  |
| Vaccinated Health Care Workers | 2.59  (0.52-12.8) | 0.24 | 1.57  (0.30-8.06) | 0.58 | 1  [Reference] |  |  | 1.81  (1.08-3.03) | ≤0.05 | 1.32  (0.70-2.49) | 0.39 | 1.11  (0.62-1.98) | 0.72 | 1  [Reference] |  |  | 1.64  (0.83-3.26) | 0.15 | 0.80  (0.44-1.44) | 0.46 | 1  [Reference] |  |  | 0.60  (0.41-0.87) | ≤0.01 | 1  [Reference] |  |  | 1.25  (0.82-1.90) | 0.28 | 1  [Reference] |  |
| Unvaccinated Health Care Workers | - | - | - | - | 1  [Reference] |  |  | 0.05  (0.01-0.24) | ≤0.01 | 0.06  (0.01-0.29) | ≤0.01 | 0.11  (0.02-0.48) | ≤0.01 | 1  [Reference] |  |  | 0.82  (0.53-1.27) | 0.37 | 1.10  (0.73-1.66) | 0.61 | 1  [Reference] |  |  | 0.73  (0.52-1.02) | 0.06 | 1  [Reference] |  |  | 0.95  (0.69-1.30) | 0.75 | 1  [Reference] |  |

**Supplement Table S1.** Univariate logistic regression analysis of factors associated with psychological outcomes among vaccinated and unvaccinated health care workers against COVID-19 infection (continued).

**Supplement Table S1.** Univariate logistic regression analysis of factors associated with psychological outcomes among vaccinated and unvaccinated health care workers against COVID-19 infection (continued).

| **Psychological outcomes** | **Providing direct service to infected patients** | | | |  | **Have you been infected with COVID-19?** | | | |  | **Have any of your family members, friends, or colleagues been infected with the COVID-19?** | | | |  | **Have any of your family members, friends, or colleagues died of the COVID-19?** | | | |  | **Have you been vaccinated against the COVID-19 infection?** | | | |  | **Social support** | | | | | | |
| --- | --- | --- | --- | --- | --- | --- | --- | --- | --- | --- | --- | --- | --- | --- | --- | --- | --- | --- | --- | --- | --- | --- | --- | --- | --- | --- | --- | --- | --- | --- | --- | --- |
|  | **Yes** | | **No** | |  | **Yes** | | **No** | |  | **Yes** | | **No** | |  | **Yes** | | **No** | |  | **Yes** | | **No** | |  | **Poor** | | **Moderate** | | **Strong** | | |
|  | **COR**  **(95% CI)** | ***p* value** | **COR**  **(95% CI)** | ***p* value** |  | **COR**  **(95% CI)** | ***p* value** | **COR**  **(95% CI)** | ***p* value** |  | **COR**  **(95% CI)** | ***p* value** | **COR**  **(95% CI)** | ***p* value** |  | **COR**  **(95% CI)** | ***p* value** | **COR**  **(95% CI)** | ***p* value** |  | **COR**  **(95% CI)** | ***p* value** | **COR**  **(95% CI)** | ***p* value** |  | **COR**  **(95% CI)** | ***p* value** | **COR**  **(95% CI)** | ***p* value** | **COR**  **(95% CI)** | ***p* value** |  |
| **General health problems** | | | | | | | | | | | | | | | | | | | | | | | | | | | | | | | |  |
| Total | 0.89  (0.74-1.06) | 0.21 | 1  [Reference] |  |  | 1.56  (1.29-1.90) | ≤0.01 | 1  [Reference] |  |  | 0.76  (0.63-0.90) | ≤0.01 | 1  [Reference] |  |  | 0.95  (0.78-1.16) | 0.66 | 1  [Reference] |  |  | 7.18  (5.83-8.84) | ≤0.01 | 1  [Reference] |  |  | 0.82  (0.62-1.07) | 0.15 | 1.17  (0.89-1.53) | 0.24 | 1  [Reference] |  |  |
| Vaccinated Health Care Workers | 0.91  (0.63-1.32) | 0.63 | 1  [Reference] |  |  | 1.30  (0.92-1.84) | 0.12 | 1  [Reference] |  |  | 0.94  (0.65-1.34) | 0.73 | 1  [Reference] |  |  | 2.67  (1.62-4.43) | ≤0.01 | 1  [Reference] |  |  | - | - | 1  [Reference] |  |  | 1.09  (0.68-1.75) | 0.70 | 1.73  (1.14-2.62) | ≤0.01 | 1  [Reference] |  |  |
| Unvaccinated Health Care Workers | 0.35  (0.27-0.45) | ≤0.01 | 1  [Reference] |  |  | 0.85  (0.64-1.14) | 0.29 | 1  [Reference] |  |  | 1.56  (1.21-2.01) | ≤0.001 | 1  [Reference] |  |  | 0.94  (0.73-1.22) | 0.68 | 1  [Reference] |  |  | - | - | 1  [Reference] |  |  | 2.33  (1.47-3.69) | ≤0.01 | 1.23  (0.76-1.99) | 0.39 | 1  [Reference] |  |  |
| **Depression symptoms** | | | | | | | | | | | | | | | | | | | | | | | | | | | | | | | |  |
| Total | 0.75  (0.61-0.92) | ≤0.01 | 1  [Reference] |  |  | 1.02  (0.82-1.27) | 0.81 | 1  [Reference] |  |  | 1.25  (1.02-1.54) | ≤0.05 | 1  [Reference] |  |  | 0.69  (0.56-0.86) | ≤0.001 | 1  [Reference] |  |  | 2.53  (2.04-3.14) | ≤0.01 | 1  [Reference] |  |  | 1.14  (0.85-1.54) | 0.36 | 1.43  (1.06-1.93) | ≤0.01 | 1  [Reference] |  |  |
| Vaccinated Health Care Workers | 0.96  (0.66-1.39) | 0.83 | 1  [Reference] |  |  | 1.00  (0.70-1.41) | 0.99 | 1  [Reference] |  |  | 1.11  (0.76-1.62) | 0.58 | 1  [Reference] |  |  | 1.52  (0.98-2.38) | 0.06 | 1  [Reference] |  |  | - | - | 1  [Reference] |  |  | 1.36  (0.82-2.24) | 0.22 | 1.63  (1.07-2.48) | ≤0.05 | 1  [Reference] |  |  |
| Unvaccinated Health Care Workers | 0.45  (0.34-0.58) | ≤0.01 | 1  [Reference] |  |  | 0.69  (0.51-0.93) | ≤0.01 | 1  [Reference] |  |  | 1.75  (1.57-2.66) | ≤0.01 | 1  [Reference] |  |  | 0.56  (0.43-0.74) | ≤0.01 | 1  [Reference] |  |  | - | - | 1  [Reference] |  |  | 1.85  (1.20-2.84) | ≤0.01 | 1.51  (0.96-2.36) | 0.07 | 1  [Reference] |  |  |
| **Anxiety symptoms** | | | | | | | | | | | | | | | | | | | | | | | | | | | | | | | |  |
| Total | 1.01  (0.83-1.24) | 0.85 | 1  [Reference] |  |  | 0.76  (0.61-0.93) | ≤0.01 | 1  [Reference] |  |  | 1.02  (0.83-1.25) | 0.81 | 1  [Reference] |  |  | 0.92  (0.74-1.14) | 0.46 | 1  [Reference] |  |  | 1.07  (0.87-1.30) | 0.50 | 1  [Reference] |  |  | 1.28  (0.94-1.74) | 0.10 | 0.92  (0.68-1.23) | 0.59 | 1  [Reference] |  |  |
| Vaccinated Health Care Workers | 0.87  (0.63-1.19) | 0.39 | 1  [Reference] |  |  | 0.90  (0.67-1.21) | 0.51 | 1  [Reference] |  |  | 0.93  (0.68-1.27) | 0.67 | 1  [Reference] |  |  | 0.79  (0.57-1.11) | 0.17 | 1  [Reference] |  |  | - | - | 1  [Reference] |  |  | 1.39  (0.89-2.16) | 0.14 | 1.24  (0.86-1.79) | 0.23 | 1  [Reference] |  |  |
| Unvaccinated Health Care Workers | 1.11  (0.84-1.47) | 0.43 | 1  [Reference] |  |  | 0.58  (0.42-0.78) | ≤0.001 | 1  [Reference] |  |  | 1.14  (0.86-1.50) | 0.34 | 1  [Reference] |  |  | 1.04  (0.77-1.39) | 0.78 | 1  [Reference] |  |  | - | - | 1  [Reference] |  |  | 1.04  (0.63-1.73) | 0.86 | 0.56  (0.33-0.94) | ≤0.05 | 1  [Reference] |  |  |
| **Stress symptoms** | | | | | | | | | | | | | | | | | | | | | | | | | | | | | | | |  |
| Total | 0.88  (0.73-1.05) | 0.17 | 1  [Reference] |  |  | 1.09  (0.90-1.33) | 0.33 | 1  [Reference] |  |  | 0.97  (0.80-1.16) | 0.75 | 1  [Reference] |  |  | 1.06  (0.87-1.30) | 0.53 | 1  [Reference] |  |  | 1.01  (0.84-1.21) | 0.89 | 1  [Reference] |  |  | 0.96  (0.72-1.26) | 0.77 | 1.04  (0.79-1.36) | 0.77 | 1  [Reference] |  |  |
| Vaccinated Health Care Workers | 0.86  (0.65-1.15) | 0.32 | 1  [Reference] |  |  | 1.20  (0.92-1.57) | 0.16 | 1  [Reference] |  |  | 1.04  (0.78-1.39) | 0.76 | 1  [Reference] |  |  | 1.23  (0.89-1.68) | 0.19 | 1  [Reference] |  |  | - | - | 1  [Reference] |  |  | 0.95  (0.64-1.43) | 0.84 | 1.01  (0.72-1.42) | 0.93 | 1  [Reference] |  |  |
| Unvaccinated Health Care Workers | 0.87  (0.67-1.12) | 0.29 | 1  [Reference] |  |  | 0.98  (0.72-1.31) | 0.89 | 1  [Reference] |  |  | 0.91  (0.71-1.18) | 0.51 | 1  [Reference] |  |  | 0.96  (0.73-1.26) | 0.79 | 1  [Reference] |  |  | - | - | 1  [Reference] |  |  | 0.97  (0.62-1.51) | 0.90 | 1.08  (0.68-1.72) | 0.72 | 1  [Reference] |  |  |
| **Post-traumatic stress disorder symptoms** | | | | | | | | | | | | | | | | | | | | | | | | | | | | | | | |  |
| Total | 0.47  (0.38-0.59) | ≤0.01 | 1  [Reference] |  |  | 0.98  (0.80-1.21) | 0.88 | 1  [Reference] |  |  | 0.93  (0.76-1.13) | 0.47 | 1  [Reference] |  |  | 0.86  (0.69-1.07) | 0.18 | 1  [Reference] |  |  | 1.54  (1.26-1.88) | ≤0.01 | 1  [Reference] |  |  | 0.42  (0.29-0.59) | ≤0.01 | 0.50  (0.35-0.70) | ≤0.01 | 1  [Reference] |  |  |
| Vaccinated Health Care Workers | 0.52  (0.36-0.74) | ≤0.01 | 1  [Reference] |  |  | 0.80  (0.59-1.08) | 0.15 | 1  [Reference] |  |  | 0.89  (0.65-1.23) | 0.51 | 1  [Reference] |  |  | 0.74  (0.53-1.04) | 0.09 | 1  [Reference] |  |  | - | - | 1  [Reference] |  |  | 0.63  (0.39-1.02) | 0.06 | 0.71  (0.47-1.08) | 0.11 | 1  [Reference] |  |  |
| Unvaccinated Health Care Workers | 0.34  (0.26-0.45) | ≤0.01 | 1  [Reference] |  |  | 0.96  (0.71-1.31) | 0.83 | 1  [Reference] |  |  | 1.16  (0.89-1.52) | 0.24 | 1  [Reference] |  |  | 1.03  (0.78-1.35) | 0.83 | 1  [Reference] |  |  | - | - | 1  [Reference] |  |  | 0.27  (0.14-0.51) | ≤0.01 | 0.26  (0.14-0.51) | ≤0.01 | 1  [Reference] |  |  |
| **Insomnia symptoms** | | | | | | | | | | | | | | | | | | | | | | | | | | | | | | | |  |
| Total | 0.87  (0.73-1.03) | 0.12 | 1  [Reference] |  |  | 1.48  (1.23-1.78) | ≤0.01 | 1  [Reference] |  |  | 0.74  (0.62-0.89) | ≤0.001 | 1  [Reference] |  |  | 0.70  (0.57-0.84) | ≤0.01 | 1  [Reference] |  |  | 5.93  (4.89-7.20) | ≤0.01 | 1  [Reference] |  |  | 0.49  (0.37-0.65) | ≤0.01 | 0.77  (0.58-1.00) | ≤0.05 | 1  [Reference] |  |  |
| Vaccinated Health Care Workers | 0.87  (0.63-1.20) | 0.39 | 1  [Reference] |  |  | 1.26  (0.93-1.69) | 0.12 | 1  [Reference] |  |  | 0.84  (0.61-1.15) | 0.27 | 1  [Reference] |  |  | 1.06  (0.74-1.50) | 0.73 | 1  [Reference] |  |  | - | - | 1  [Reference] |  |  | 0.61  (0.39-0.97) | ≤0.05 | 0.79  (0.53-1.18) | 0.25 | 1  [Reference] |  |  |
| Unvaccinated Health Care Workers | 0.32  (0.24-0.42) | ≤0.01 | 1  [Reference] |  |  | 0.78  (0.58-1.06) | 0.11 | 1  [Reference] |  |  | 1.60  (1.23-2.08) | ≤0.01 | 1  [Reference] |  |  | 0.70  (0.53-0.92) | ≤0.01 | 1  [Reference] |  |  | - | - | 1  [Reference] |  |  | 1.24  (0.79-1.93) | 0.34 | 0.87  (0.54-1.39) | 0.57 | 1  [Reference] |  |  |
| **Loneliness symptoms** | | | | | | | | | | | | | | | | | | | | | | | | | | | | | | | |  |
| Total | 0.63  (0.50-0.80) | ≤0.01 | 1  [Reference] |  |  | 1.33  (1.04-1.71) | ≤0.05 | 1  [Reference] |  |  | 0.75  (0.60-0.95) | ≤0.01 | 1  [Reference] |  |  | 1.16  (0.89-1.49) | 0.25 | 1  [Reference] |  |  | 1.73  (1.37-2.18) | ≤0.01 | 1  [Reference] |  |  | 0.43  (0.29-0.63) | ≤0.01 | 0.66  (0.44-0.99) | ≤0.05 | 1  [Reference] |  |  |
| Vaccinated Health Care Workers | 0.72  (0.48-1.09) | 0.12 | 1  [Reference] |  |  | 1.54  (1.06-2.25) | ≤0.05 | 1  [Reference] |  |  | 0.72  (0.49-1.05) | ≤0.05 | 1  [Reference] |  |  | 1.10  (0.71-1.70) | 0.65 | 1  [Reference] |  |  | - | - | 1  [Reference] |  |  | 0.48  (0.28-0.83) | ≤0.01 | 1.06  (0.64-1.75) | 0.79 | 1  [Reference] |  |  |
| Unvaccinated Health Care Workers | 0.45  (0.33-0.61) | ≤0.01 | 1  [Reference] |  |  | 0.94  (0.67-1.32) | 0.73 | 1  [Reference] |  |  | 0.96  (0.72-1.30) | 0.83 | 1  [Reference] |  |  | 1.32  (0.96-1.81) | 0.08 | 1  [Reference] |  |  | - | - | 1  [Reference] |  |  | 0.36  (0.18-0.71) | ≤0.01 | 0.36  (0.18-0.72) | ≤0.01 | 1  [Reference] |  |  |

Abbreviation: COR, Crude odds ratio; CI, confidence interval.

**Supplement Table S2.** Multivariate logistic regression analysis of factors associated with psychological outcomes among vaccinated and unvaccinated health care workers against COVID-19 infection.

| **Psychological outcomes** | **Sex** | | | | | |  | **Age, y** | | | | | | | |  | **Residence** | | | |  | **Marital status** | | | | | |  | **Having children** | | | |
| --- | --- | --- | --- | --- | --- | --- | --- | --- | --- | --- | --- | --- | --- | --- | --- | --- | --- | --- | --- | --- | --- | --- | --- | --- | --- | --- | --- | --- | --- | --- | --- | --- |
|  | **Male** | | **Female** | | **Not interested** | |  | **18-29** | | **30-39** | | **40-49** | | **≥50** | |  | **Urban** | | **Rural** | |  | **Single** | | **Married** | | **Divorced/separated/widowed** | |  | **Yes** | | **No** | |
|  | **AOR**  **(95% CI)** | ***p* value** | **AOR**  **(95% CI)** | ***p* value** | **AOR**  **(95% CI)** | ***p* value** |  | **AOR**  **(95% CI)** | ***p* value** | **AOR**  **(95% CI)** | ***p* value** | **AOR**  **(95% CI)** | ***p* value** | **AOR**  **(95% CI)** | ***p* value** |  | **AOR**  **(95% CI)** | ***p* value** | **AOR**  **(95% CI)** | ***p* value** |  | **AOR**  **(95% CI)** | ***p* value** | **AOR**  **(95% CI)** | ***p* value** | **AOR**  **(95% CI)** | ***p* value** |  | **AOR**  **(95% CI)** | ***p* value** | **AOR**  **(95% CI)** | ***p* value** |
| **General health problems** | | | | | | | | | | | | | | | | | | | | | | | | | | | | | | | | |
| Total | 1.52  (0.74-3.11) | 0.25 | 1.33  (0.65-2.72) | 0.43 | 1  [Reference] |  |  | 1.65  (0.63-4.31) | 0.30 | 1.20  (0.52-2.79) | 0.66 | 1.22  (0.61-2.44) | 0.56 | 1  [Reference] |  |  | 0.43  (0.33-0.55) | ≤0.01 | 1  [Reference] |  |  | 0.41  (0.23-0.74) | ≤0.01 | 0.39  (0.25-0.61) | ≤0.01 | 1  [Reference] |  |  | 1.21  (0.81-1.81) | 0.33 | 1  [Reference] |  |
| Vaccinated Health Care Workers | 2.68  (0.96-7.43) | ≤0.05 | 2.71  (0.97-7.60) | ≤0.05 | 1  [Reference] |  |  | 1.55  (0.18-12.7) | ≤0.01 | 0.78  (0.15-3.93) | ≤0.01 | 0.75  (0.23-2.47) | ≤0.01 | 1  [Reference] |  |  | 0.15  (0.09-0.25) | ≤0.01 | 1  [Reference] |  |  | 0.06  (0.01-0.41) | ≤0.01 | 0.10  (0.02-0.39) | ≤0.001 | 1  [Reference] |  |  | 0.66  (0.25-1.71) | 0.39 | 1  [Reference] |  |
| Unvaccinated Health Care Workers | 2.36  (0.45-12.1) | 0.30 | 1.86  (0.35-9.64) | 0.46 | 1  [Reference] |  |  | 1.27  (0.40-4.05) | ≤0.01 | 0.94  (0.33-2.65) | 0.91 | 1.26  (0.51-3.09) | 0.61 | 1  [Reference] |  |  | 0.84  (0.62-1.14) | 0.28 | 1  [Reference] |  |  | 1.00  (0.51-1.97) | 0.97 | 1.05  (0.63-1.74) | 0.84 | 1  [Reference] |  |  | 1.22  (0.75-1.98) | 0.40 | 1  [Reference] |  |
| **Depression symptoms** | | |  | | | | | | | | | | | |  | | | | | | | | | | | | | | | | | |
| Total | 0.46  (0.20-1.09) | 0.08 | 0.52  (0.22-1.23) | 0.13 | 1  [Reference] |  |  | 0.89  (0.35-2.22) | 0.80 | 1.63  (0.74-3.57) | 0.22 | 1.77  (0.94-3.31) | 0.07 | 1  [Reference] |  |  | 0.71  (0.56-0.91) | ≤0.01 | 1  [Reference] |  |  | 1.32  (0.75-2.34) | 0.32 | 0.67  (0.44-1.01) | ≤0.05 | 1  [Reference] |  |  | 0.97  (0.64-1.47) | 0.90 | 1  [Reference] |  |
| Vaccinated Health Care Workers | - | - | - | - | 1  [Reference] |  |  | 1.44  (0.07-2.57) | ≤0.01 | 1.51  (0.31-5.68) | ≤0.01 | 1.84  (0.63-5.37) | 0.06 | 1  [Reference] |  |  | 0.43  (0.27-0.67) | ≤0.01 | 1  [Reference] |  |  | 0.42  (0.12-1.44) | 0.16 | 0.31  (0.22-0.82) | ≤0.01 | 1  [Reference] |  |  | 0.53  (0.23-1.21) | 0.13 | 1  [Reference] |  |
| Unvaccinated Health Care Workers | - | - | - | - | 1  [Reference] |  |  | 1.83  (0.27-2.60) | ≤0.01 | 1.34  (0.50-3.58) | ≤0.05 | 1.21  (0.52-2.78) | ≤0.05 | 1  [Reference] |  |  | 1.15  (0.83-1.59) | 0.38 | 1  [Reference] |  |  | 2.60  (1.29-5.22) | ≤0.01 | 1.18  (0.71-1.95) | 0.50 | 1  [Reference] |  |  | 1.10  (0.65-1.85) | 0.70 | 1  [Reference] |  |
| **Anxiety symptoms** | | | | | | | | | | | | | | | | | | | | | | | | | | | | | | | | |
| Total | 1.49  (0.82-2.69) | 0.18 | 1.57  (0.87-2.84) | 0.13 | 1  [Reference] |  |  | 0.56  (0.22-1.39) | 0.21 | 0.72  (0.32-1.58) | 0.41 | 0.92  (0.50-1.69) | 0.79 | 1  [Reference] |  |  | 0.83  (0.65-1.05) | 0.12 | 1  [Reference] |  |  | 1.50  (0.87-2.57) | 0.14 | 1.14  (0.76-1.70) | 0.50 | 1  [Reference] |  |  | 0.99  (0.68-1.45) | 0.98 | 1  [Reference] |  |
| Vaccinated Health Care Workers | 1.82  (0.97-3.44) | 0.06 | 2.17  (1.14-4.13) | ≤0.01 | 1  [Reference] |  |  | - | - | - | - | - | - | 1  [Reference] |  |  | 0.77  (0.54-1.08) | 0.13 | 1  [Reference] |  |  | - | - | - | - | 1  [Reference] |  |  | - | - | 1  [Reference] |  |
| Unvaccinated Health Care Workers | 0.48  (0.05-4.11) | 0.50 | 0.50  (0.05-4.30) | 0.53 | 1  [Reference] |  |  | - | - | - | - | - | - | 1  [Reference] |  |  | 0.90  (0.65-1.23) | 0.52 | 1  [Reference] |  |  | - | - | - | - | 1  [Reference] |  |  | - | - | 1  [Reference] |  |
| **Stress symptoms** | | | | | | | | | | | | | | | | | | | | | | | |  | | | | | | | | |
| Total | 1.47  (0.85-2.54) | 0.16 | 1.16  (0.67-2.01) | 0.59 | 1  [Reference] |  |  | 1.16  (0.51-2.64) | 0.72 | 0.97  (0.47-2.01) | 0.95 | 1.46  (0.82-2.59) | 0.19 | 1  [Reference] |  |  | 0.77  (0.62-0.96) | ≤0.05 | 1  [Reference] |  |  | 1.00  (0.61-1.65) | 0.98 | 0.89  (0.61-1.28) | 0.54 | 1  [Reference] |  |  | 1.15  (0.81-1.61) | 0.42 | 1  [Reference] |  |
| Vaccinated Health Care Workers | - | - | - | - | 1  [Reference] |  |  | 1.07  (0.33-3.46) | 0.90 | 1.16  (0.39-3.49) | 0.78 | 1.15  (0.53-2.52) | 0.71 | 1  [Reference] |  |  | 0.64  (0.47-0.88) | ≤0.01 | 1  [Reference] |  |  | - | - | - | - | 1  [Reference] |  |  | - | - | 1  [Reference] |  |
| Unvaccinated Health Care Workers | - | - | - | - | 1  [Reference] |  |  | 1.87  (0.33-2.29) | ≤0.01 | 0.67  (0.27-1.65) | 0.39 | 1.28  (0.59-2.80) | 0.08 | 1  [Reference] |  |  | 0.91  (0.68-1.22) | 0.55 | 1  [Reference] |  |  | - | - | - | - | 1  [Reference] |  |  | - | - | 1  [Reference] |  |
| **Post-traumatic stress disorder symptoms** | | | | | | | | | | | | | | | | | | | | | | | | | | | | | | | | |
| Total | 1.75  (0.90-3.43) | 0.09 | 1.65  (0.84-3.22) | 0.14 | 1  [Reference] |  |  | 0.35  (0.11-1.08) | 0.07 | 0.57  (0.20-1.60) | 0.28 | 1.54  (0.61-3.87) | 0.35 | 1  [Reference] |  |  | 0.75  (0.58-0.96) | ≤0.05 | 1  [Reference] |  |  | 0.57  (0.29-1.13) | 0.10 | 0.62  (0.35-1.13) | 0.11 | 1  [Reference] |  |  | 0.81  (0.55-1.19) | 0.28 | 1  [Reference] |  |
| Vaccinated Health Care Workers | 1.39  (0.67-2.89) | 0.37 | 1.56  (0.75-3.26) | 0.22 | 1  [Reference] |  |  | 0.25  (0.04-1.41) | 0.12 | 0.46  (0.09-2.21) | ≤0.05 | 0.67  (0.21-2.18) | 0.51 | 1  [Reference] |  |  | - | - | 1  [Reference] |  |  | 0.62  (0.21-1.80) | 0.38 | 0.78  (0.31-1.96) | 0.60 | 1  [Reference] |  |  | 0.78  (0.42-1.47) | 0.45 | 1  [Reference] |  |
| Unvaccinated Health Care Workers | 2.74  (0.40-18.7) | 0.30 | 2.24  (0.32-15.4) | 0.41 | 1  [Reference] |  |  | 1.35  (0.08-1.48) | ≤0.01 | 0.56  (0.15-2.09) | ≤0.05 | 2.10  (0.59-7.42) | 0.24 | 1  [Reference] |  |  | - | - | 1  [Reference] |  |  | 0.50  (0.21-1.20) | 0.12 | 0.59  (0.28-1.26) | 0.17 | 1  [Reference] |  |  | 0.78  (0.46-1.32) | 0.36 | 1  [Reference] |  |
| **Insomnia symptoms** | | | | | | | | | | | | | | | | | | | | | | | | | | | | | | | | |
| Total | 0.92  (0.48-1.77) | 0.82 | 0.80  (0.41-1.53) | 0.50 | 1  [Reference] |  |  | 0.74  (0.30-1.81) | 0.51 | 1.21  (0.55-2.67) | 0.62 | 1.17  (0.61-2.22) | 0.63 | 1  [Reference] |  |  | 0.67  (0.53-0.84) | ≤0.001 | 1  [Reference] |  |  | 0.83  (0.49-1.43) | 0.51 | 0.73  (0.49-1.09) | 0.12 | 1  [Reference] |  |  | 0.87  (0.60-1.05) | 0.26 | 1  [Reference] |  |
| Vaccinated Health Care Workers | 1.16  (0.53-2.53) | 0.69 | 0.79  (0.36-1.71) | 0.54 | 1  [Reference] |  |  | 2.96  (0.67-12.9) | 0.06 | 4.46  (1.21-16.4) | ≤0.05 | 2.57  (0.98-6.60) | ≤0.05 | 1  [Reference] |  |  | 0.41  (0.29-0.59) | ≤0.01 | 1  [Reference] |  |  | 0.37  (0.13-1.01) | ≤0.05 | 0.46  (0.20-1.03) | ≤0.05 | 1  [Reference] |  |  | 0.65  (0.35-1.20) | 0.17 | 1  [Reference] |  |
| Unvaccinated Health Care Workers | 1.01  (0.19-5.24) | 0.98 | 0.98  (0.18-5.07) | 0.96 | 1  [Reference] |  |  | 0.41  (0.13-1.27) | 0.12 | 0.63  (0.23-1.73) | 0.37 | 0.65  (0.27-1.55) | 0.34 | 1  [Reference] |  |  | 0.99  (0.73-1.34) | 0.97 | 1  [Reference] |  |  | 1.43  (0.73-2.79) | 0.29 | 1.14  (0.68-1.89) | 0.60 | 1  [Reference] |  |  | 0.91  (0.56-1.47) | 0.70 | 1  [Reference] |  |
| **Loneliness symptoms** | | | | | | | | |  | | | | | | | | | | | | | | | | | | | | | | | |
| Total | 2.96  (1.52-5.78) | ≤0.001 | 2.76  (1.41-5.38) | ≤0.01 | 1  [Reference] |  |  | 0.35  (0.11-1.09) | 0.07 | 0.26  (0.09-0.70) | ≤0.01 | 0.46  (0.20-1.06) | 0.06 | 1  [Reference] |  |  | 0.58  (0.44-0.76) | ≤0.01 | 1  [Reference] |  |  | 0.21  (0.09-0.46) | ≤0.01 | 0.24  (0.12-0.47) | ≤0.01 | 1  [Reference] |  |  | 0.94  (0.62-1.44) | 0.80 | 1  [Reference] |  |
| Vaccinated Health Care Workers | 1.97  (0.88-4.43) | 0.09 | 2.52  (1.11-5.73) | ≤0.05 | 1  [Reference] |  |  | 1.57  (0.10-3.10) | 0.13 | 0.47  (0.11-2.01) | 0.31 | 1.44  (0.15-1.23) | 0.12 | 1  [Reference] |  |  | 0.29  (0.19-0.44) | ≤0.01 | 1  [Reference] |  |  | 0.43  (0.11-1.64) | 0.22 | 0.31  (0.10-0.92) | ≤0.05 | 1  [Reference] |  |  | 0.78  (0.38-1.59) | ≤0.01 | 1  [Reference] |  |
| Unvaccinated Health Care Workers | 7.62  (1.17-49.6) | ≤0.05 | 5.69  (0.87-37.0) | 0.06 | 1  [Reference] |  |  | 1.23  (0.03-1.72) | ≤0.01 | 1.16  (0.02-1.05) | ≤0.05 | 0.50  (0.08-1.32) | 0.46 | 1  [Reference] |  |  | 1.04  (0.72-1.50) | 0.80 | 1  [Reference] |  |  | 0.24  (0.08-0.68) | ≤0.01 | 0.51  (0.20-1.28) | 0.15 | 1  [Reference] |  |  | 1.07  (0.62-1.84) | 0.06 | 1  [Reference] |  |

| **Psychological outcomes** | **Highest education level** | | | | | | | |  | **Working position** | | | | | | | | | |  | **Work types** | | | |  | **Employment titles** | | | |
| --- | --- | --- | --- | --- | --- | --- | --- | --- | --- | --- | --- | --- | --- | --- | --- | --- | --- | --- | --- | --- | --- | --- | --- | --- | --- | --- | --- | --- | --- |
|  | **Bachelor (MBBS) or lower degree** | | **Post-graduate degree** | | **Doctoral degree** | | **Other** | |  | **Doctor** | | **Nurse** | | **Medical technician** | | **Hospital workers** | | **Other** | |  | **Frontline** | | **Second-line** | |  | **Senior** | | **Intermediate** | |
|  | **AOR**  **(95% CI)** | ***p* value** | **AOR**  **(95% CI)** | ***p* value** | **AOR**  **(95% CI)** | ***p* value** | **AOR**  **(95% CI)** | ***p* value** |  | **AOR**  **(95% CI)** | ***p* value** | **AOR**  **(95% CI)** | ***p* value** | **AOR**  **(95% CI)** | ***p* value** | **AOR**  **(95% CI)** | ***p* value** | **AOR**  **(95% CI)** | ***p***  **value** |  | **AOR**  **(95% CI)** | ***p***  **value** | **AOR**  **(95% CI)** | ***p***  **value** |  | **AOR**  **(95% CI)** | ***p***  **value** | **AOR**  **(95% CI)** | ***p***  **value** |
| **General health problems** | | | | | | | | | | | | | | | | | | | | | | | | |  | | | | |
| Total | 26.2  (3.84-179.2) | ≤0.001 | 15.6  (2.31-105.4) | ≤0.01 | 17.0  (2.46-117.6) | ≤0.01 | 1  [Reference] |  |  | 0.39  (0.28-0.53) | ≤0.01 | 0.51  (0.32-0.82) | ≤0.01 | 0.69  (0.47-1.00) | ≤0.05 | 0.60  (0.42-0.85) | ≤0.01 | 1  [Reference] |  |  | 0.61  (0.41-0.90) | ≤0.01 | 1  [Reference] |  |  | 8.36  (0.98-70.6) | ≤0.05 | 22.6  (2.65-193.0) | ≤0.01 |
| Vaccinated Health Care Workers | 42.6  (3.67-495.6) | ≤0.01 | 24.9  (2.22-281.0) | ≤0.01 | 29.3  (2.46-348.4) | ≤0.01 | 1  [Reference] |  |  | 0.18  (0.08-0.37) | ≤0.01 | 0.22  (0.07-0.69) | ≤0.01 | 0.38  (0.13-1.15) | 0.08 | 0.49  (0.17-1.42) | 0.19 | 1  [Reference] |  |  | 0.74  (0.38-1.46) | 0.06 | 1  [Reference] |  |  | 1.37  (0.14-13.0) | 0.78 | 5.06  (0.50-50.7) | 0.16 |
| Unvaccinated Health Care Workers | - | - | - | - | - | - | 1  [Reference] |  |  | 0.70  (0.47-1.03) | 0.07 | 0.71  (0.41-1.22) | 0.22 | 0.80  (0.53-1.21) | 0.29 | 0.67  (0.45-1.00) | ≤0.05 | 1  [Reference] |  |  | 1.65  (0.38-1.10) | ≤0.01 | 1  [Reference] |  |  | - | - | - | - |
| **Depression symptoms** | | | | | | | | | | | | | | | | | | | | | | | | |  | | | | |
| Total | 5.84  (1.23-27.7) | ≤0.05 | 6.76  (1.44-31.7) | ≤0.01 | 5.29  (1.11-25.2) | ≤0.05 | 1  [Reference] |  |  | 1.04  (0.77-1.40) | 0.79 | 0.97  (0.62-1.52) | 0.90 | 1.35  (0.91-2.00) | 0.12 | 1.27  (0.88-1.83) | 0.18 | 1  [Reference] |  |  | 0.56  (0.38-0.82) | ≤0.01 | 1  [Reference] |  |  | 2.05  (0.55-7.67) | 0.28 | 2.42  (0.65-9.03) | 0.18 |
| Vaccinated Health Care Workers | 7.66  (1.09-53.6) | ≤0.05 | 7.16  (1.03-49.6) | ≤0.05 | 6.16  (0.84-44.7) | 0.07 | 1  [Reference] |  |  | 0.51  (0.30-0.87) | ≤0.01 | 0.81  (0.32-2.06) | 0.66 | 1.17  (0.44-3.05) | 0.74 | 0.55  (0.26-1.16) | 0.11 | 1  [Reference] |  |  | 1.80  (0.47-1.36) | 0.41 | 1  [Reference] |  |  | 0.62  (0.14-2.74) | 0.53 | 1.99  (0.44-9.03) | 0.36 |
| Unvaccinated Health Care Workers | - | - | - | - | - | - | 1  [Reference] |  |  | 1.98  (1.32-3.06) | ≤0.001 | 1.44  (0.84-2.46) | ≤0.05 | 1.60  (1.02-2.52) | ≤0.05 | 1.88  (1.21-2.90) | ≤0.01 | 1  [Reference] |  |  | 2.41  (0.23-3.73) | ≤0.01 | 1  [Reference] |  |  | 3.78  (0.21-67.5) | 0.36 | 1.50  (0.08-26.6) | 0.78 |
| **Anxiety symptoms** | | | | | | | | | | | | | | | | | | | | | | | | |  | | | | |
| Total | 1.13  (0.21-6.07) | 0.88 | 0.96  (0.18-5.09) | 0.96 | 1.08  (0.20-5.83) | 0.92 | 1  [Reference] |  |  | 0.56  (0.42-0.74) | ≤0.01 | 0.53  (0.35-0.80) | ≤0.01 | 0.70  (0.49-1.00) | ≤0.05 | 0.87  (0.61-1.23) | 0.43 | 1  [Reference] |  |  | 0.93  (0.67-1.30) | 0.69 | 1  [Reference] |  |  | 2.01  (0.56-7.15) | 0.27 | 2.34  (0.66-8.29) | 0.18 |
| Vaccinated Health Care Workers | - | - | - | - | - | - | 1  [Reference] |  |  | 0.54  (0.37-0.78) | ≤0.001 | 1.84  (0.44-1.61) | 0.60 | 0.91  (0.49-1.70) | 0.77 | 0.90  (0.51-1.60) | 0.74 | 1  [Reference] |  |  | - | - | 1  [Reference] |  |  | - | - | 1.82  (0.42-7.76) | - |
| Unvaccinated Health Care Workers | - | - | - | - | - | - | 1  [Reference] |  |  | 1.72  (0.48-1.06) | 0.10 | 1.42  (0.24-1.73) | ≤0.01 | 0.59  (0.38-0.92) | ≤0.05 | 0.81  (0.52-1.25) | 0.35 | 1  [Reference] |  |  | - | - | 1  [Reference] |  |  | - | - | 3.15  (0.17-57.9) | - |
| **Stress symptoms** | | | | | | | | | | | | | | | | | | | | | | | | |  | | | | |
| Total | 1.00  (0.22-4.85) | 0.99 | 1.24  (0.28-5.46) | 0.77 | 1.00  (0.22-4.47) | 0.99 | 1  [Reference] |  |  | 1.13  (0.87-1.47) | 0.32 | 0.79  (0.54-1.15) | 0.22 | 1.09  (0.79-1.52) | 0.57 | 0.86  (0.63-1.16) | 0.33 | 1  [Reference] |  |  | 0.78  (0.57-1.06) | 0.12 | 1  [Reference] |  |  | 0.35  (0.07-1.75) | 0.20 | 0.29  (0.06-1.44) | 0.13 |
| Vaccinated Health Care Workers | - | - | - | - | - | - | 1  [Reference] |  |  | 1.24  (0.85-1.80) | 0.25 | 1.33  (0.72-2.46) | 0.34 | 1.28  (0.72-2.28) | 0.38 | 0.78  (0.47-1.28) | 0.33 | 1  [Reference] |  |  | 0.77  (0.58-1.02) | 0.07 | 1  [Reference] |  |  | - | - | - | - |
| Unvaccinated Health Care Workers | - | - | - | - | - | - | 1  [Reference] |  |  | 1.19  (0.80-1.75) | 0.37 | 1.55  (0.31-0.89) | ≤0.05 | 1.08  (0.71-1.63) | ≤0.01 | 0.86  (0.57-1.28) | 0.46 | 1  [Reference] |  |  | 0.86  (0.66-1.12) | 0.28 | 1  [Reference] |  |  | - | - | - | - |
| **Post-traumatic stress disorder symptoms** | | | | | | | | | | | | | | |  |  | | | | | | | | |  | | | | |
| Total | - | - | - | - | - | - | 1  [Reference] |  |  | 0.98  (0.73-1.32) | 0.92 | 1.04  (0.68-1.59) | 0.85 | 0.94  (0.65-1.36) | 0.76 | 1.22  (0.85-1.75) | 0.26 | 1  [Reference] |  |  | 0.69  (0.48-0.99) | ≤0.05 | 1  [Reference] |  |  | 1.45  (0.15-13.8) | 0.74 | 0.50  (0.05-4.48) | 0.53 |
| Vaccinated Health Care Workers | - | - | - | - | - | - | 1  [Reference] |  |  | - | - | - | - | - | - | - | - | 1  [Reference] |  |  | 1.80  (0.52-2.23) | ≤0.05 | 1  [Reference] |  |  | - | - | - | - |
| Unvaccinated Health Care Workers | - | - | - | - | - | - | 1  [Reference] |  |  | - | - | - | - | - | - | - | - | 1  [Reference] |  |  | 1.75  (0.41-2.12) | ≤0.01 | 1  [Reference] |  |  | - | - | - | - |
| **Insomnia symptoms** | | | | | | | | | | | | | | | | | | | | | | | | |  | | | | |
| Total | 2.08  (0.45-9.58) | 0.34 | 2.09  (0.46-9.49) | 0.33 | 2.12  (0.46-9.76) | 0.33 | 1  [Reference] |  |  | 1.08  (0.82-1.42) | 0.57 | 0.58  (0.38-0.89) | ≤0.01 | 0.81  (0.58-1.14) | 0.23 | 0.93  (0.67-1.28) | 0.65 | 1  [Reference] |  |  | 0.63  (0.45-0.88) | ≤0.01 | 1  [Reference] |  |  | 1.07  (0.27-4.26) | 0.92 | 1.96  (0.49-7.76) | 0.33 |
| Vaccinated Health Care Workers | - | - | - | - | - | - | 1  [Reference] |  |  | 0.75  (0.50-1.13) | 0.17 | 0.50  (0.26-0.95) | ≤0.05 | 0.36  (0.20-0.65) | ≤0.001 | 1.00  (0.53-1.87) | 0.99 | 1  [Reference] |  |  | 0.81  (0.53-2.25) | 0.35 | 1  [Reference] |  |  | - | - | - | - |
| Unvaccinated Health Care Workers | - | - | - | - | - | - | 1  [Reference] |  |  | 1.60  (1.09-2.35) | 0.20 | 0.65  (0.35-1.22) | 0.18 | 1.15  (0.77-1.73) | 0.47 | 1.02  (0.69-1.50) | 0.90 | 1  [Reference] |  |  | 2.45  (0.26-3.78) | ≤0.01 | 1  [Reference] |  |  | - | - | - | - |
| **Loneliness symptoms** | | | | | | | | | | | | | | | | | | | | | | | | |  | | | | |
| Total | 1.98  (0.31-12.4) | 0.46 | 1.87  (0.29-11.7) | 0.50 | 2.28  (0.35-14.7) | 0.38 | 1  [Reference] |  |  | 1.10  (0.79-1.53) | 0.56 | 0.86  (0.54-1.36) | 0.52 | 1.51  (0.96-2.37) | 0.06 | 1.15  (0.77-1.71) | 0.49 | 1  [Reference] |  |  | 1.50  (0.33-0.76) | ≤0.001 | 1  [Reference] |  |  | 0.56  (0.10-3.14) | 0.51 | 1.26  (0.23-6.83) | 0.78 |
| Vaccinated Health Care Workers | - | - | - | - | - | - | 1  [Reference] |  |  | 0.83  (0.51-1.36) | 0.47 | 0.72  (0.32-1.61) | 0.42 | 2.03  (0.73-5.59) | 0.17 | 1.37  (0.61-3.06) | 0.43 | 1  [Reference] |  |  | 1.47  (0.26-0.85) | ≤0.01 | 1  [Reference] |  |  | - | - | - | - |
| Unvaccinated Health Care Workers | - | - | - | - | - | - | 1  [Reference] |  |  | 1.63  (1.04-2.54) | 0.86 | 1.16  (0.66-2.05) | 0.59 | 1.47  (0.88-2.46) | 0.13 | 1.15  (0.73-1.83) | 0.53 | 1  [Reference] |  |  | 1.59  (0.31-1.92) | ≤0.01 | 1  [Reference] |  |  | - | - | - | - |

**Supplement Table S2.** Multivariate logistic regression analysis of factors associated with psychological outcomes among vaccinated and unvaccinated health care workers against COVID-19 infection (continued).

| **Psychological outcomes** |  | | | | | |  | **Work experiences, y** | | | | | | | |  | **Socio economic status** | | | | | |  | **Living with family** | | | |  | **Smoking habit** | | | |
| --- | --- | --- | --- | --- | --- | --- | --- | --- | --- | --- | --- | --- | --- | --- | --- | --- | --- | --- | --- | --- | --- | --- | --- | --- | --- | --- | --- | --- | --- | --- | --- | --- |
|  | **Junior** | | **New** | | **Other** | |  | **≤5** | | **6-10** | | **11-19** | | **≥20** | |  | **Lower class** | | **Middle class** | | **Upper class** | |  | **Yes** | | **No** | |  | **Yes** | | **No** | |
|  | **AOR**  **(95% CI)** | ***p* value** | **AOR**  **(95% CI)** | ***p* value** | **AOR**  **(95% CI)** | ***p* value** |  | **AOR**  **(95% CI)** | ***p* value** | **AOR**  **(95% CI)** | ***p* value** | **AOR**  **(95% CI)** | ***p* value** | **AOR**  **(95% CI)** | ***p* value** |  | **AOR**  **(95% CI)** | ***p* value** | **AOR**  **(95% CI)** | ***p* value** | **AOR**  **(95% CI)** | ***p* value** |  | **AOR**  **(95% CI)** | ***p* value** | **AOR**  **(95% CI)** | ***p* value** |  | **AOR**  **(95% CI)** | ***p* value** | **AOR**  **(95% CI)** | ***p* value** |
| **General health problems** | | | | |  | | | | | | | | | | | | | | | | | | | | | | | | | | | |
| Total | 23.7  (2.69-208.5) | ≤0.01 | 21.3  (2.37-192.8) | ≤0.01 | 1  [Reference] |  |  | 1.22  (0.46-3.22) | 0.68 | 0.97  (0.39-2.37) | 0.94 | 0.82  (0.40-1.69) | 0.60 | 1  [Reference] |  |  | 1.11  (0.75-1.63) | 0.58 | 1.12  (0.81-1.55) | 0.48 | 1  [Reference] |  |  | 0.87  (0.68-1.11) | 0.27 | 1  [Reference] |  |  | 0.92  (0.71-1.20) | 0.56 | 1  [Reference] |  |
| Vaccinated Health Care Workers | 21.9  (1.95-244.9) | ≤0.01 | 12.2  (0.95-157.3) | ≤0.05 | 1  [Reference] |  |  | 0.47  (0.07-3.08) | ≤0.01 | 0.32  (0.06-1.74) | ≤0.01 | 0.88  (0.28-2.76) | ≤0.05 | 1  [Reference] |  |  | 1.52  (0.59-3.93) | 0.38 | 0.88  (0.43-1.78) | 0.72 | 1  [Reference] |  |  | 0.57  (0.32-1.04) | 0.06 | 1  [Reference] |  |  | 0.78  (0.43-1.40) | 0.41 | 1  [Reference] |  |
| Unvaccinated Health Care Workers | - | - | - | - | 1  [Reference] |  |  | 1.21  (0.35-4.20) | ≤0.05 | 1.02  (0.32-3.22) | 0.23 | 0.82  (0.30-2.21) | 0.70 | 1  [Reference] |  |  | 1.15  (0.73-1.80) | 0.54 | 1.32  (0.90-1.94) | 0.15 | 1  [Reference] |  |  | 1.23  (0.91-1.66) | 0.17 | 1  [Reference] |  |  | 1.01  (0.75-1.36) | 0.91 | 1  [Reference] |  |
| **Depression symptoms** | | | | | | | | | | | | | | | | | | | | | | | | | | | | | | | | |
| Total | 2.61  (0.67-10.1) | 0.16 | 2.70  (0.67-10.8) | 0.15 | 1  [Reference] |  |  | 1.29  (0.51-3.26) | 0.58 | 1.23  (0.53-2.84) | 0.62 | 0.95  (0.50-1.83) | 0.89 | 1  [Reference] |  |  | 1.10  (0.75-1.61) | 0.61 | 0.99  (0.72-1.36) | 0.98 | 1  [Reference] |  |  | 0.83  (0.65-1.07) | 0.16 | 1  [Reference] |  |  | 1.11  (0.85-1.44) | 0.42 | 1  [Reference] |  |
| Vaccinated Health Care Workers | 1.92  (0.38-9.57) | 0.42 | 2.35  (0.42-13.0) | 0.32 | 1  [Reference] |  |  | 0.93  (0.16-5.28) | ≤0.01 | 0.83  (0.17-4.10) | ≤0.01 | 0.44  (0.14-1.29) | 0.08 | 1  [Reference] |  |  | 1.33  (0.62-2.87) | 0.45 | 0.89  (0.48-1.63) | 0.71 | 1  [Reference] |  |  | 1.10  (0.69-1.75) | 0.67 | 1  [Reference] |  |  | - | - | 1  [Reference] |  |
| Unvaccinated Health Care Workers | 1.77  (0.10-31.3) | 0.69 | 1.84  (0.10-33.2) | 0.67 | 1  [Reference] |  |  | 2.20  (0.64-7.54) | ≤0.05 | 2.23  (0.73-6.79) | ≤0.05 | 2.48  (0.96-6.36) | ≤0.05 | 1  [Reference] |  |  | 1.02  (0.64-1.62) | 0.92 | 1.07  (0.72-1.57) | 0.73 | 1  [Reference] |  |  | 0.83  (0.60-1.15) | 0.27 | 1  [Reference] |  |  | - | - | 1  [Reference] |  |
| **Anxiety symptoms** | | | | | | | | | | | | | | | | | | | | | | | | | | | | | | | | |
| Total | 1.98  (0.54-7.20) | 0.29 | 1.66  (0.44-6.21) | 0.45 | 1  [Reference] |  |  | 1.80  (0.74-4.35) | 0.19 | 1.36  (0.60-3.08) | 0.45 | 1.35  (0.73-2.49) | 0.32 | 1  [Reference] |  |  | 1.26  (0.88-1.80) | 0.19 | 1.25  (0.92-1.69) | 0.14 | 1  [Reference] |  |  | 1.05  (0.84-1.32) | 0.63 | 1  [Reference] |  |  | 0.86  (0.68-1.10) | 0.24 | 1  [Reference] |  |
| Vaccinated Health Care Workers | - | - | - | - | 1  [Reference] |  |  | 1.36  (0.82-2.24) | ≤0.05 | 0.76  (0.42-1.36) | 0.36 | 1.44  (0.84-2.47) | ≤0.05 | 1  [Reference] |  |  | 0.61  (0.33-1.12) | 0.11 | 0.66  (0.39-1.13) | 0.13 | 1  [Reference] |  |  | - | - | 1  [Reference] |  |  | - | - | 1  [Reference] |  |
| Unvaccinated Health Care Workers | - | - | - | - | 1  [Reference] |  |  | 1.16  (0.66-2.04) | 0.58 | 1.34  (0.74-2.42) | 0.32 | 1.30  (0.74-2.28) | 0.34 | 1  [Reference] |  |  | 1.86  (1.18-2.94) | ≤0.01 | 1.77  (1.21-2.59) | ≤0.01 | 1  [Reference] |  |  | - | - | 1  [Reference] |  |  | - | - | 1  [Reference] |  |
| **Stress symptoms** | | | |  | | | | | | | | | | | | | | | | | | | | | | | | | | | | |
| Total | 0.47  (0.09-2.32) | 0.35 | 0.45  (0.09-2.31) | 0.34 | 1  [Reference] |  |  | 1.21  (0.53-2.73) | 0.64 | 1.05  (0.50-2.23) | 0.88 | 1.14  (0.64-2.03) | 0.65 | 1  [Reference] |  |  | 0.91  (0.65-1.28) | 0.61 | 0.97  (0.73-1.30) | 0.87 | 1  [Reference] |  |  | 1.03  (0.84-1.27) | 0.73 | 1  [Reference] |  |  | 0.69  (0.55-0.86) | ≤0.001 | 1  [Reference] |  |
| Vaccinated Health Care Workers | - | - | - | - | 1  [Reference] |  |  | 0.89  (0.29-2.71) | 0.84 | 0.69  (0.23-2.02) | 0.49 | 0.83  (0.39-1.77) | 0.63 | 1  [Reference] |  |  | - | - | - | - | 1  [Reference] |  |  | - | - | 1  [Reference] |  |  | 0.80  (0.60-1.08) | 0.15 | 1  [Reference] |  |
| Unvaccinated Health Care Workers | - | - | - | - | 1  [Reference] |  |  | 2.37  (0.93-6.07) | ≤0.05 | 1.96  (0.80-4.78) | 0.13 | 1.53  (0.71-3.28) | 0.26 | 1  [Reference] |  |  | - | - | - | - | 1  [Reference] |  |  | - | - | 1  [Reference] |  |  | 0.71  (0.53-0.94) | ≤0.01 | 1  [Reference] |  |
| **Post-traumatic stress disorder symptoms** | | | | | | | | | | | | | | | | | | | | | | | | | | | | | | |  | |
| Total | 0.42  (0.04-3.83) | 0.44 | 0.29  (0.03-2.74) | 0.28 | 1  [Reference] |  |  | 0.89  (0.28-2.81) | 0.84 | 0.94  (0.31-2.83) | 0.92 | 0.68  (0.27-1.75) | 0.43 | 1  [Reference] |  |  | 1.40  (0.96-2.03) | 0.07 | 1.58  (1.13-2.21) | ≤0.01 | 1  [Reference] |  |  | 0.83  (0.65-1.05) | 0.12 | 1  [Reference] |  |  | 0.91  (0.71-1.17) | 0.48 | 1  [Reference] |  |
| Vaccinated Health Care Workers | - | - | - | - | 1  [Reference] |  |  | 0.80  (0.17-3.65) | 0.77 | 1.01  (0.22-4.49) | 0.98 | 1.07  (0.35-3.23) | 0.90 | 1  [Reference] |  |  | - | - | - | - | 1  [Reference] |  |  | - | - | 1  [Reference] |  |  | 0.85  (0.61-1.20) | 0.38 | 1  [Reference] |  |
| Unvaccinated Health Care Workers | - | - | - | - | 1  [Reference] |  |  | 0.11  (0.02-0.59) | ≤0.01 | 0.13  (0.02-0.70) | ≤0.01 | 0.10  (0.02-0.50) | ≤0.01 | 1  [Reference] |  |  | - | - | - | - | 1  [Reference] |  |  | - | - | 1  [Reference] |  |  | 0.81  (0.57-1.14) | 0.23 | 1  [Reference] |  |
| **Insomnia symptoms** | | | | | | | | | | | | | | | | | | | | | | | | | | | | | | | | |
| Total | 2.35  (0.57-9.54) | 0.23 | 2.55  (0.61-10.6) | 0.19 | 1  [Reference] |  |  | 0.61  (0.24-1.50) | 0.28 | 0.59  (0.25-1.35) | 0.21 | 0.66  (0.34-1.28) | 0.22 | 1  [Reference] |  |  | 1.09  (0.77-1.56) | 0.60 | 1.03  (0.76-1.40) | 0.82 | 1  [Reference] |  |  | 0.85  (0.68-1.05) | 0.14 | 1  [Reference] |  |  | 1.20  (0.95-1.52) | 0.11 | 1  [Reference] |  |
| Vaccinated Health Care Workers | - | - | - | - | 1  [Reference] |  |  | 0.51  (0.13-2.02) | 0.07 | 0.28  (0.07-1.07) | 0.06 | 0.55  (0.21-1.42) | 0.19 | 1  [Reference] |  |  | - | - | - | - | 1  [Reference] |  |  | 0.86  (0.61-1.21) | 0.39 | 1  [Reference] |  |  | - | - | 1  [Reference] |  |
| Unvaccinated Health Care Workers | - | - | - | - | 1  [Reference] |  |  | 1.12  (0.38-3.25) | 0.82 | 1.34  (0.49-3.65) | 0.55 | 1.26  (0.54-2.96) | 0.58 | 1  [Reference] |  |  | - | - | - | - | 1  [Reference] |  |  | 0.86  (0.64-1.15) | 0.32 | 1  [Reference] |  |  | - | - | 1  [Reference] |  |
| **Loneliness symptoms** | | | | | | | | | | | | | | | | | | | | | | | | | | | | | | | | |
| Total | 1.04  (0.19-5.70) | 0.95 | 0.58  (0.10-3.26) | 0.53 | 1  [Reference] |  |  | 1.15  (0.38-3.45) | 0.79 | 1.07  (0.38-2.94) | 0.89 | 0.81  (0.36-1.81) | 0.61 | 1  [Reference] |  |  | 1.23  (0.80-1.88) | 0.33 | 1.07  (0.74-1.54) | 0.70 | 1  [Reference] |  |  | 0.64  (0.49-0.85) | ≤0.01 | 1  [Reference] |  |  | 1.06  (0.80-1.42) | 0.65 | 1  [Reference] |  |
| Vaccinated Health Care Workers | - | - | - | - | 1  [Reference] |  |  | 0.95  (0.21-4.22) | 0.09 | 1.22  (0.29-5.08) | 0.16 | 1.21  (0.45-3.26) | 0.19 | 1  [Reference] |  |  | - | - | - | - | 1  [Reference] |  |  | 0.82  (0.52-1.29) | 0.41 | 1  [Reference] |  |  | - | - | 1  [Reference] |  |
| Unvaccinated Health Care Workers | - | - | - | - | 1  [Reference] |  |  | 0.30  (0.04-2.11) | ≤0.05 | 0.34  (0.05-2.27) | ≤0.01 | 0.31  (0.05-1.91) | ≤0.01 | 1  [Reference] |  |  | - | - | - | - | 1  [Reference] |  |  | 0.63  (0.43-0.91) | ≤0.01 | 1  [Reference] |  |  | - | - | 1  [Reference] |  |

**Supplement Table S2.** Multivariate logistic regression analysis of factors associated with psychological outcomes among vaccinated and unvaccinated health care workers against COVID-19 infection (continued).

**Supplement Table S2.** Multivariate logistic regression analysis of factors associated with psychological outcomes among vaccinated and unvaccinated health care workers against COVID-19 infection (continued).

| **Psychological outcomes** | **Providing direct service to infected patients** | | | |  | **Have you been infected with COVID-19?** | | | |  | **Have any of your family members, friends, or colleagues been infected with the COVID-19?** | | | |  | **Have any of your family members, friends, or colleagues died of the COVID-19?** | | | |  | **Have you been vaccinated against the COVID-19 infection?** | | | |  | **Social support** | | | | | | |
| --- | --- | --- | --- | --- | --- | --- | --- | --- | --- | --- | --- | --- | --- | --- | --- | --- | --- | --- | --- | --- | --- | --- | --- | --- | --- | --- | --- | --- | --- | --- | --- | --- |
|  | **Yes** | | **No** | |  | **Yes** | | **No** | |  | **Yes** | | **No** | |  | **Yes** | | **No** | |  | **Yes** | | **No** | |  | **Poor** | | **Moderate** | | **Strong** | | |
|  | **AOR**  **(95% CI)** | ***p* value** | **AOR**  **(95% CI)** | ***p* value** |  | **AOR**  **(95% CI)** | ***p* value** | **AOR**  **(95% CI)** | ***p* value** |  | **AOR**  **(95% CI)** | ***p* value** | **AOR**  **(95% CI)** | ***p* value** |  | **AOR**  **(95% CI)** | ***p* value** | **AOR**  **(95% CI)** | ***p* value** |  | **AOR**  **(95% CI)** | ***p* value** | **AOR**  **(95% CI)** | ***p* value** |  | **AOR**  **(95% CI)** | ***p* value** | **AOR**  **(95% CI)** | ***p* value** | **AOR**  **(95% CI)** | ***p* value** |  |
| **General health problems** | | | | | | | | | | | | | | | | | | | | | | | | | | | | | | | |  |
| Total | 0.75  (0.50-1.11) | 0.16 | 1  [Reference] |  |  | 1.13  (0.88-1.45) | ≤0.01 | 1  [Reference] |  |  | 0.92  (0.71-1.19) | 0.54 | 1  [Reference] |  |  | 1.04  (0.81-1.32) | 0.75 | 1  [Reference] |  |  | 15.8  (11.5-21.7) | ≤0.01 | 1  [Reference] |  |  | 1.35  (0.91-2.00) | 0.13 | 1.01  (0.69-1.46) | 0.94 | 1  [Reference] |  |  |
| Vaccinated Health Care Workers | 1.19  (0.59-2.41) | 0.61 | 1  [Reference] |  |  | 1.44  (0.88-2.38) | 0.14 | 1  [Reference] |  |  | 0.71  (0.36-1.38) | 0.32 | 1  [Reference] |  |  | 1.54  (0.90-2.65) | 0.11 | 1  [Reference] |  |  | - | - | 1  [Reference] |  |  | 0.93  (0.44-1.93) | 0.84 | 1.10  (0.60-2.03) | 0.74 | 1  [Reference] |  |  |
| Unvaccinated Health Care Workers | 1.54  (0.31-2.93) | ≤0.05 | 1  [Reference] |  |  | 0.89  (0.65-1.22) | ≤0.05 | 1  [Reference] |  |  | 0.99  (0.74-1.33) | 0.97 | 1  [Reference] |  |  | 0.83  (0.62-1.11) | 0.22 | 1  [Reference] |  |  | - | - | 1  [Reference] |  |  | 1.69  (0.97-2.95) | 0.06 | 0.94  (0.54-1.64) | 0.84 | 1  [Reference] |  |  |
| **Depression symptoms** | | | | | | | | | | | | | | | | | | | | | | | | | | | | | | | |  |
| Total | 1.03  (0.70-1.52) | 0.86 | 1  [Reference] |  |  | 0.82  (0.64-1.04) | 0.11 | 1  [Reference] |  |  | 1.36  (1.07-1.73) | ≤0.01 | 1  [Reference] |  |  | 0.73  (0.57-0.94) | ≤0.01 | 1  [Reference] |  |  | 3.30  (2.49-4.38) | ≤0.01 | 1  [Reference] |  |  | 1.13  (0.79-1.62) | 0.48 | 1.16  (0.83-1.63) | 0.37 | 1  [Reference] |  |  |
| Vaccinated Health Care Workers | 1.25  (0.72-2.17) | 0.42 | 1  [Reference] |  |  | 0.96  (0.65-1.43) | 0.87 | 1  [Reference] |  |  | 0.93  (0.56-1.52) | 0.77 | 1  [Reference] |  |  | 1.00  (0.64-1.54) | 0.99 | 1  [Reference] |  |  | - | - | 1  [Reference] |  |  | 1.33  (0.75-2.35) | 0.32 | 1.35  (0.84-2.18) | 0.21 | 1  [Reference] |  |  |
| Unvaccinated Health Care Workers | 1.06  (0.59-1.90) | ≤0.01 | 1  [Reference] |  |  | 0.66  (0.48-0.92) | ≤0.01 | 1  [Reference] |  |  | 1.55  (1.15-2.09) | ≤0.01 | 1  [Reference] |  |  | 0.55  (0.41-0.75) | ≤0.01 | 1  [Reference] |  |  | - | - | 1  [Reference] |  |  | 1.18  (0.68-2.03) | 0.55 | 1.06  (0.62-1.81) | 0.81 | 1  [Reference] |  |  |
| **Anxiety symptoms** | | | | | | | | | | | | | | | | | | | | | | | | | | | | | | | |  |
| Total | 1.11  (0.79-1.57) | 0.52 | 1  [Reference] |  |  | 0.74  (0.59-0.92) | ≤0.01 | 1  [Reference] |  |  | 1.11  (0.88-1.40) | 0.35 | 1  [Reference] |  |  | 1.09  (0.86-1.36) | 0.45 | 1  [Reference] |  |  | 1.25  (0.97-1.62) | 0.07 | 1  [Reference] |  |  | 1.36  (0.97-1.92) | 0.07 | 0.91  (0.67-1.24) | 0.57 | 1  [Reference] |  |  |
| Vaccinated Health Care Workers | - | - | 1  [Reference] |  |  | 0.89  (0.66-1.20) | 0.45 | 1  [Reference] |  |  | - | - | 1  [Reference] |  |  | - | - | 1  [Reference] |  |  | - | - | 1  [Reference] |  |  | 1.49  (0.94-2.35) | 0.08 | 1.23  (0.84-1.79) | 0.27 | 1  [Reference] |  |  |
| Unvaccinated Health Care Workers | - | - | 1  [Reference] |  |  | 0.63  (0.46-0.87) | ≤0.01 | 1  [Reference] |  |  | - | - | 1  [Reference] |  |  | - | - | 1  [Reference] |  |  | - | - | 1  [Reference] |  |  | 0.88  (0.49-1.57) | 0.67 | 0.51  (0.29-0.90) | ≤0.05 | 1  [Reference] |  |  |
| **Stress symptoms** | | | | | | | | | | | | | | | | | | | | | | | | | | | | | | | |  |
| Total | 1.06  (0.77-1.45) | 0.69 | 1  [Reference] |  |  | 1.11  (0.91-1.37) | 0.28 | 1  [Reference] |  |  | 0.92  (0.75-1.14) | 0.48 | 1  [Reference] |  |  | 1.03  (0.84-1.27) | 0.71 | 1  [Reference] |  |  | 0.94  (0.75-1.19) | 0.65 | 1  [Reference] |  |  | 0.97  (0.71-1.32) | 0.85 | 1.04  (0.78-1.39) | 0.74 | 1  [Reference] |  |  |
| Vaccinated Health Care Workers | - | - | 1  [Reference] |  |  | - | - | 1  [Reference] |  |  | - | - | 1  [Reference] |  |  | - | - | 1  [Reference] |  |  | - | - | 1  [Reference] |  |  | - | - | - | - | 1  [Reference] |  |  |
| Unvaccinated Health Care Workers | - | - | 1  [Reference] |  |  | - | - | 1  [Reference] |  |  | - | - | 1  [Reference] |  |  | - | - | 1  [Reference] |  |  | - | - | 1  [Reference] |  |  | - | - | - | - | 1  [Reference] |  |  |
| **Post-traumatic stress disorder symptoms** | | | | | | | | | | | | | | | | | | | | | | | | | | | | | | | |  |
| Total | 0.43  (0.29-0.62) | ≤0.01 | 1  [Reference] |  |  | 0.83  (0.65-1.06) | 0.13 | 1  [Reference] |  |  | 1.10  (0.86-1.40) | 0.42 | 1  [Reference] |  |  | 1.00  (0.79-1.28) | 0.94 | 1  [Reference] |  |  | 2.06  (1.55-2.73) | ≤0.01 | 1  [Reference] |  |  | 0.81  (0.55-1.21) | 0.32 | 0.76  (0.52-1.11) | 0.15 | 1  [Reference] |  |  |
| Vaccinated Health Care Workers | 0.61  (0.38-1.98) | ≤0.05 | 1  [Reference] |  |  | - | - | 1  [Reference] |  |  | - | - | 1  [Reference] |  |  | - | - | 1  [Reference] |  |  | - | - | 1  [Reference] |  |  | 0.79  (0.48-1.30) | 0.39 | 0.87  (0.56-1.34) | 0.54 | 1  [Reference] |  |  |
| Unvaccinated Health Care Workers | 1.29  (0.16-1.53) | ≤0.01 | 1  [Reference] |  |  | - | - | 1  [Reference] |  |  | - | - | 1  [Reference] |  |  | - | - | 1  [Reference] |  |  | - | - | 1  [Reference] |  |  | 0.70  (0.33-1.50) | 0.36 | 0.55  (0.26-1.18) | 0.12 | 1  [Reference] |  |  |
| **Insomnia symptoms** | | | | | | | | | | | | | | | | | | | | | | | | | | | | | | | |  |
| Total | 0.65  (0.46-0.92) | ≤0.01 | 1  [Reference] |  |  | 1.02  (0.81-1.27) | 0.84 | 1  [Reference] |  |  | 1.25  (0.99-1.56) | ≤0.05 | 1  [Reference] |  |  | 1.00  (0.79-1.25) | 0.99 | 1  [Reference] |  |  | 7.39  (5.70-9.57) | ≤0.01 | 1  [Reference] |  |  | 0.81  (0.57-1.14) | 0.23 | 0.72  (0.52-1.00) | ≤0.05 | 1  [Reference] |  |  |
| Vaccinated Health Care Workers | 2.95  (0.60-1.49) | 0.83 | 1  [Reference] |  |  | - | - | 1  [Reference] |  |  | 1.11  (0.76-1.62) | 0.55 | 1  [Reference] |  |  | 1.37  (0.97-1.93) | 0.07 | 1  [Reference] |  |  | - | - | 1  [Reference] |  |  | 0.63  (0.38-1.03) | 0.06 | 0.68  (0.44-1.05) | 0.08 | 1  [Reference] |  |  |
| Unvaccinated Health Care Workers | 2.60  (0.34-3.06) | ≤0.05 | 1  [Reference] |  |  | - | - | 1  [Reference] |  |  | 1.33  (0.98-1.80) | 0.06 | 1  [Reference] |  |  | 0.77  (0.56-1.04) | 0.09 | 1  [Reference] |  |  | - | - | 1  [Reference] |  |  | 1.00  (0.58-1.73) | 0.98 | 0.72  (0.42-1.25) | 0.25 | 1  [Reference] |  |  |
| **Loneliness symptoms** | | | | | | | | | | | | | | | | | | | | | | | | | | | | | | | |  |
| Total | 0.84  (0.55-1.28) | 0.42 | 1  [Reference] |  |  | 1.12  (0.86-1.47) | 0.38 | 1  [Reference] |  |  | 0.86  (0.65-1.13) | 0.29 | 1  [Reference] |  |  | 1.15  (0.88-1.50) | 0.29 | 1  [Reference] |  |  | 1.73  (1.28-2.34) | ≤0.01 | 1  [Reference] |  |  | 0.59  (0.38-0.93) | ≤0.05 | 0.77  (0.50-1.18) | 0.24 | 1  [Reference] |  |  |
| Vaccinated Health Care Workers | 1.16  (0.64-2.08) | 0.62 | 1  [Reference] |  |  | 1.56  (1.04-2.34) | ≤0.05 | 1  [Reference] |  |  | 1.13  (0.70-1.83) | 0.60 | 1  [Reference] |  |  | - | - | 1  [Reference] |  |  | - | - | 1  [Reference] |  |  | 0.53  (0.30-0.95) | ≤0.05 | 0.64  (0.35-1.18) | 0.15 | 1  [Reference] |  |  |
| Unvaccinated Health Care Workers | 2.56  (0.30-3.06) | ≤0.01 | 1  [Reference] |  |  | 0.80  (0.55-1.17) | 0.26 | 1  [Reference] |  |  | 0.71  (0.50-1.01) | ≤0.05 | 1  [Reference] |  |  | - | - | 1  [Reference] |  |  | - | - | 1  [Reference] |  |  | 1.07  (0.47-2.44) | 0.86 | 0.94  (0.58-1.52) | 0.80 | 1  [Reference] |  |  |

Abbreviation: AOR, Adjusted odds ratio; CI, confidence interval.
